# Nanostructurally Controllable Strong Wood Aerogel toward Efficient Thermal Insulation

**DOI:** 10.1021/acsami.2c04584

**Published:** 2022-05-05

**Authors:** Jonas Garemark, Jesus E. Perea-Buceta, Daniel Rico del Cerro, Stephen Hall, Barbara Berke, Ilkka Kilpeläinen, Lars A. Berglund, Yuanyuan Li

**Affiliations:** † Wallenberg Wood Science Center, Department of Fiber and Polymer Technology, KTH Royal Institute of Technology, SE-10044 Stockholm, Sweden; ‡ Department of Chemistry, University of Helsinki, A.I. Virtasen aukio 1, 00560 Helsinki, Finland; § Lund University, Division of Solid Mechanics, SE-221 00 Lund, Sweden; ∥ Department of Physics, Chalmers University of Technology, 412 96 Gothenburg, Sweden

**Keywords:** aerogel, wood, ionic liquid, thermal insulation, sustainable materials

## Abstract

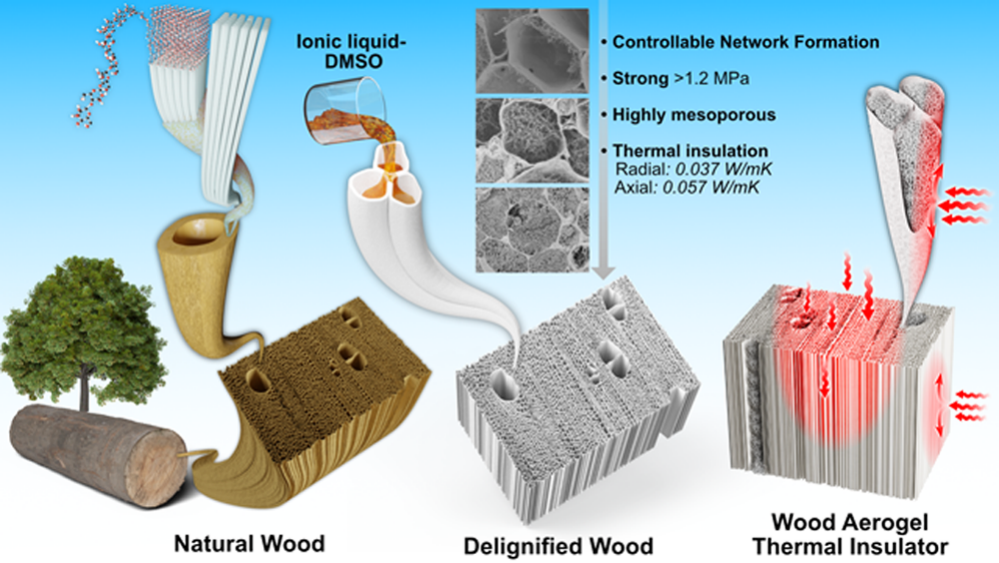

Eco-friendly materials with superior thermal insulation and mechanical properties are desirable for improved energy- and space-efficiency in buildings. Cellulose aerogels with structural anisotropy could fulfill these requirements, but complex processing and high energy demand are challenges for scaling up. Here we propose a scalable, nonadditive, top-down fabrication of strong anisotropic aerogels directly from wood with excellent, near isotropic thermal insulation functions. The aerogel was obtained through cell wall dissolution and controlled precipitation in lumen, using an ionic liquid (IL) mixture comprising DMSO and a guanidinium phosphorus-based IL [MTBD][MMP]. The wood aerogel shows a unique structure with lumen filled with nanofibrils network. In situ formation of a cellulosic nanofibril network in the lumen results in specific surface areas up to 280 m^2^/g and high yield strengths >1.2 MPa. The highly mesoporous structure (average pore diameter ∼20 nm) of freeze-dried wood aerogels leads to low thermal conductivities in both the radial (0.037 W/mK) and axial (0.057 W/mK) directions, showing great potential as scalable thermal insulators. This synthesis route is energy efficient with high nanostructural controllability. The unique nanostructure and rare combination of strength and thermal properties set the material apart from comparable bottom-up aerogels. This nonadditive synthesis approach is believed to contribute significantly toward large-scale design and structure control of biobased aerogels.

## Introduction

Heating and cooling of buildings results in one of our largest carbon footprints, accounting for over 10% of the global CO_2_ emissions.^1^ Developing mechanically strong and high performing thermal insulators with low embodied energy is crucial to energy savings in buildings and sustainable development. Aerogels with structural anisotropy have significant prospects in this context, as their ultralight (density 0.001–0.2 g/cm^3^) and porous frameworks (porosity ≥90%) can combine low thermal conductivities with high strength.^2^

Mainstream silica aerogels suffer from brittleness, scale-up, and sustainability issues.^3,4^ Anisotropic aerogels from nonbrittle, renewable precursors such as cellulose can overcome these limitations. Aerogels from bottom-up strategy using nanocellulose or dissolved cellulose is prominent in the literature.^5−7^ But complex processing and high energy consumption are issues for scale-up production. Wood is a low cost and eco-friendly cellulose composite with an already existing molecular prearrangement. Top-down synthesis, exploiting the natural hierarchical and anisotropic native wood (NW) structure, can provide high-performance anisotropic aerogels. One category of wood aerogels is reported based on delignification and hemicellulose removal from wood.^8−10^ This type of wood aerogels show low specific surface area (<50 m^2^/g) and empty fiber lumen (diameter ∼30 μm), which leads to worse thermal insulation in the axial direction, limiting their roles in advanced applications. The problem can be solved by introduction of nanoporosity in the lumen space, reducing the mean free path of air.^11^ Introducing extra polymer networks into lumen has been demonstrated, yet the homogeneous infiltration and scaling-up production are challenges.^12,13^ Previously, we reported a method to generate nanoporosity in the lumen space through partial cell wall dissolution in DMAc/LiCl followed by regenerating nanofibril networks in lumen.^14^ The limitations lie in the moisture sensitivity of DMAc/LiCl for cell wall dissolution and inferior structural controllability of nanocellulosic networks formation. In this context, ionic liquids (ILs) are a promising alternative.^15,16^ With 10^18^ possible combinations of cations and anions, ILs can be designed with controlled reaction ability with wood.^17^ In the literature, ILs have been reported to dissolve wood powders, while limited attention is on wood veneer/block treatment.^18,19^ Rarely reported is in situ nanofibril network formation in wood lumen.

**Figure 1:**
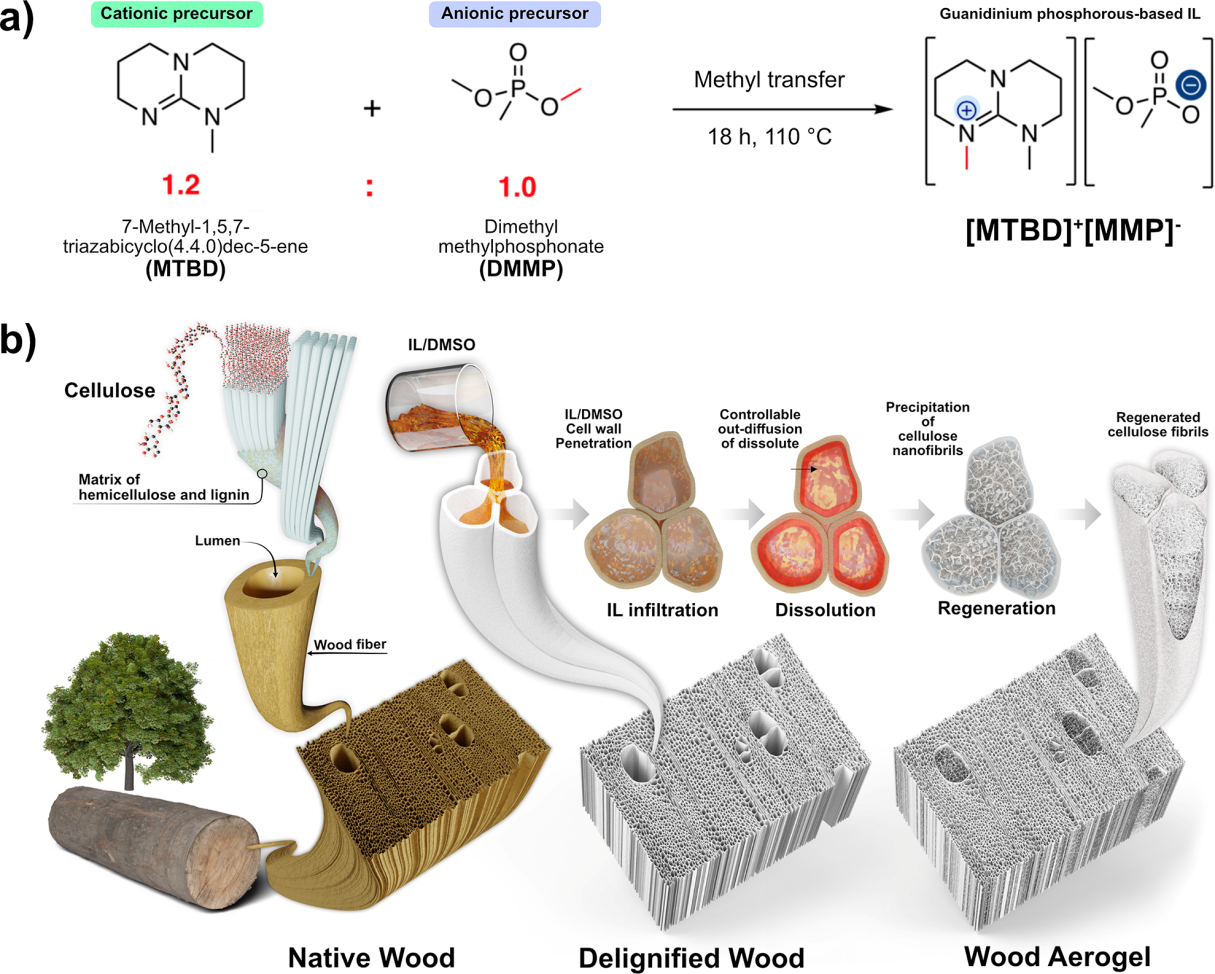
(a) Preparation of [MTBD]^+^[MMP]^−^; (b) Schematic of wood aerogels formation. The left-hand side illustrates the hierarchical structure of wood. From the delignified wood, depiction of the IL penetration, cellulosic dissolution, and cellulosic precipitation are shown. The final wood aerogel shows lumen space filled with nanofibrils networks.

In this work, nanostructurally controllable wood aerogels with nanofibrils filling the lumen were prepared through a top-down synthesis by partial delignification and a guanidinium phosphorus-based IL ([MTBD]^+^[MMP]^−^) treatment (Figure 1). Structure control of the cellulosic nanofibrils network in lumen could be achieved by tailoring the cell wall dissolution and mass diffusion. The nanofibril network-filling wood aerogel structure leads to a unique combination of high strength (∼1.2 MPa) and high specific surface area (up to 280 m^2^/g) and low thermal conductivities both in axial (0.057 W/mK) and radial (0.037 W/mK) directions.

## Results and Discussion

To obtain the wood aerogel with nanostructural control, ILs were designed to meet the requirements. ILs with phosphonate-based anions, and guanidinium-based cations were investigated for wood processing. After reactivity test and parameters screening using Enocell cellulose (low hemicellulose content) and balsa wood (see Supporting Information (SI) Figures S1 and S2 for full details), the guanidinium phosphorus-based IL ([MTBD]^+^[MMP]^−^) was selected for wood aerogel fabrication (Figure 1). DMSO was added (optimal IL:DMSO ratio was 20:80 wt %) to decrease viscosity and support dissolution. The IL [MTBD]^+^[MMP]^−^ has a molar ratio of 1.2:1; while [MTBD] excess stabilizes the IL. Detailed nuclear magnetic resonance (NMR) analysis of [MTBD]^+^[MMP]^−^ can be seen in SI Figures S3–S7. The ^31^P NMR analysis of IL (SI Figure S7) showed the chemical stability after 5 months of storage. TGA and DSC results showed a high thermal stability of IL up to ∼260 °C (SI Figure S8).

**Figure 2:**
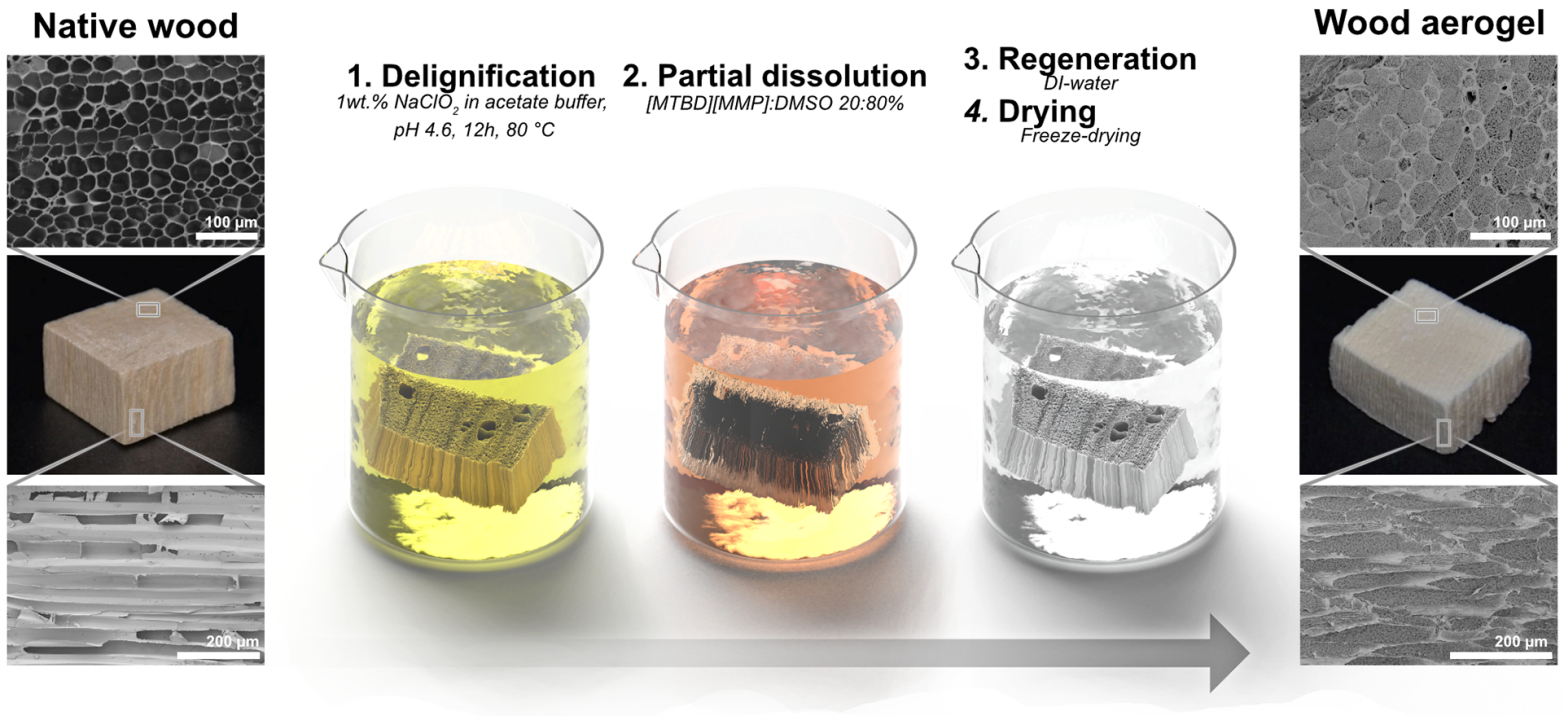
Synthesis of wood aerogels. Top left-hand side, Native wood (NW) exhibits its neighboring fibers with empty centers (up) and bottom image shows the long empty fibers in the axial direction of wood. The following are illustrations of NW treatment. In the upper right-hand side image, nanofibril networks homogeneously fill the fiber centers in wood. Bottom right-hand side image shows complete filling of all fibers in axial direction of the wood aerogel.

Figure 2 shows the fabrication procedure for wood aerogels with controllable nanofibril networks. Representative microscopic structure of native wood (anisotropic structure with empty lumen) and wood aerogels (anisotropic structure with cellulosic nanofibril network filling in the lumen) are shown. In general, four steps are involved (1) delignification to make the cell wall more permeable and accessible by generation of nanoporosity in the cell wall;^20^ (2) IL/DMSO treatment of the activated DW, in which the cell wall is partially dissolved and diffuses into the lumen; (3) Precipitation of dissolved material by addition of water, in which intermolecular forces provide stability and nanofibrillated networks are formed in the empty fiber lumen and results in hydrogels; and (4) Drying of the gel-form substrates using freeze-drying (FD) or critical point drying (CPD).

**Figure 3:**
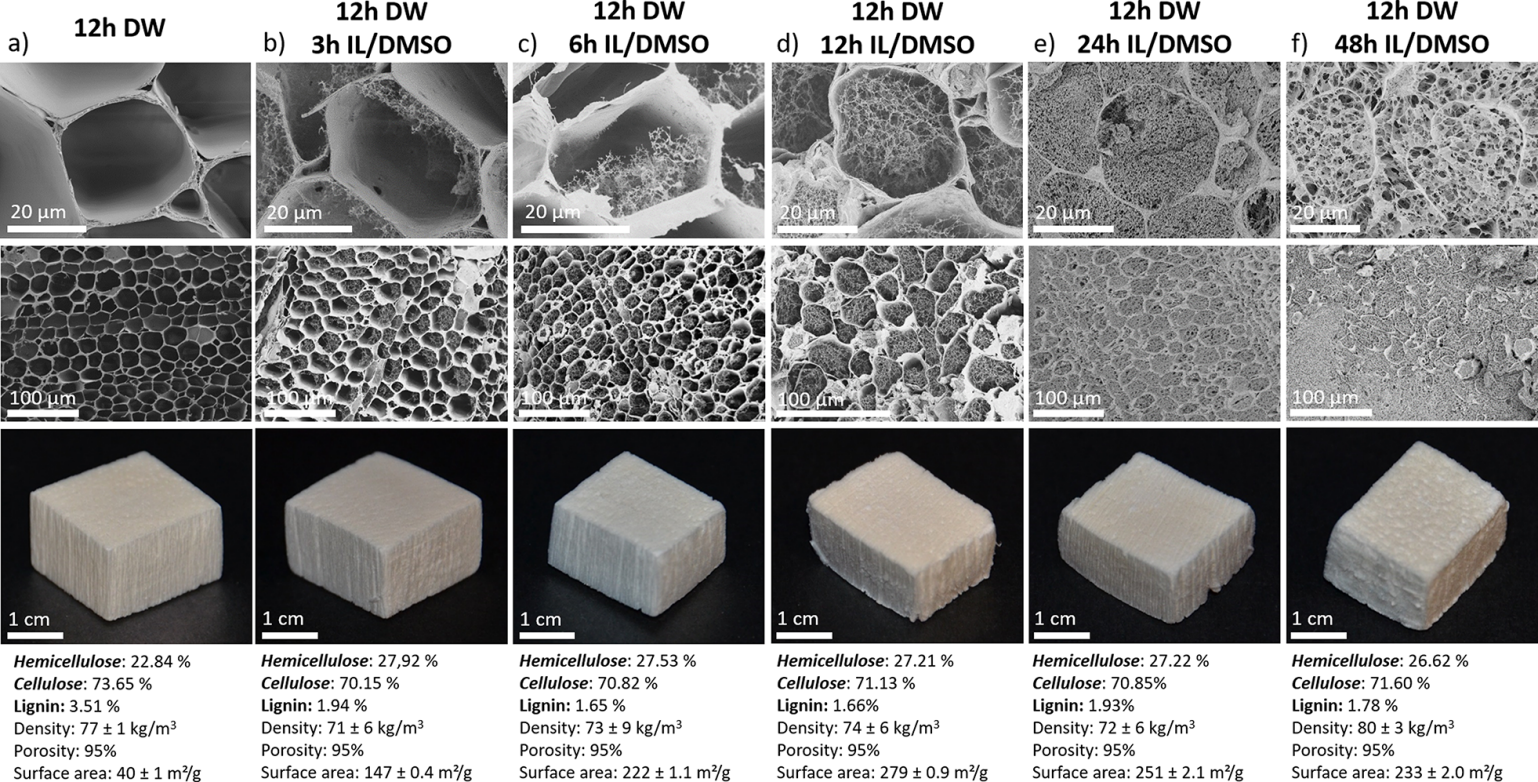
SEM of cross sections of DW after 12 h delignification (a), followed by IL treatment of 12h DW for (b) 3 h, (c) 6 h, (d) 12 h, (e) 24 h, and (f) 48 h. The second row shows lower magnification SEM images of homogeneous structures. The third row represents the wood specimen from which the SEM images were obtained.

Figure 3 shows controlled cellulosic nanofibril network formation in the lumen. Partial delignification was performed to obtain aerogels with good mechanical properties. Twelve h delignification was selected, yielding DW with 3.5 wt % lignin, whereas the starting NW had an initial lignin content of 22 wt %. DW preserves the native wood macrostructure (Figures 3a and 2). Following delignification, the IL-treatment was performed. The temporal evolution of the wood aerogel under IL-treatment is apparent in Figure 3b–f. Nanofibril networks within the lumen developed with treatment time and cell wall thinning was clear in the process, providing an opportunity for nanostructural control. At short treatment time (3 h), a “forest-like” architecture advanced from the lumen/cell wall interface. This progressed until 12 h, when partially filled lumen were observed. Beyond 24 h, all the microscale pore-space in the fiber cross sections were completely filled by nanofibrillar structures, including the cell wall corners. These structures exhibit excellent homogeneity in longitudinal, transverse, and radial planes, see SI Figure S9. High magnification SEM images of lumen fibrillar structures can be seen in SI Figure S10. Low magnification SEM images are shown in SI Figure S11 to illustrate this homogeneity. The samples from the final treatment time (48 h) showed a thin cell wall, surrounded by nanofibrils, see Figure 3f. Throughout, the wood aerogel exhibits the hierarchical structure of wood. This structure control was achieved due to favorable dissolution kinetics and controllable diffusion of the dissolute, which was not attainable in previous report using DMAc/LiCl.^14^ Materials yield decreased gradually with IL treatment, 18 wt % loss after delignification and 52 wt % loss after 48 h IL treatment (relative to NW). The weight loss is ascribed to delignification and cellulose/hemicellulose diffusion to the solution bath during IL treatment. A slight increase in wood aerogel density was noticed compared to DW (Figure 3). This is mainly due to the shrinkage of the samples during regeneration. Images of sample dimensional changes are seen in SI Figure S12, while detailed data of dimensional changes and weight loss are summarized in SI Table S1. Larger specimens could also be achieved, SI Figure S13 show samples of 1.5, 2.5, and 6 cm in specimen width.

**Figure 4:**
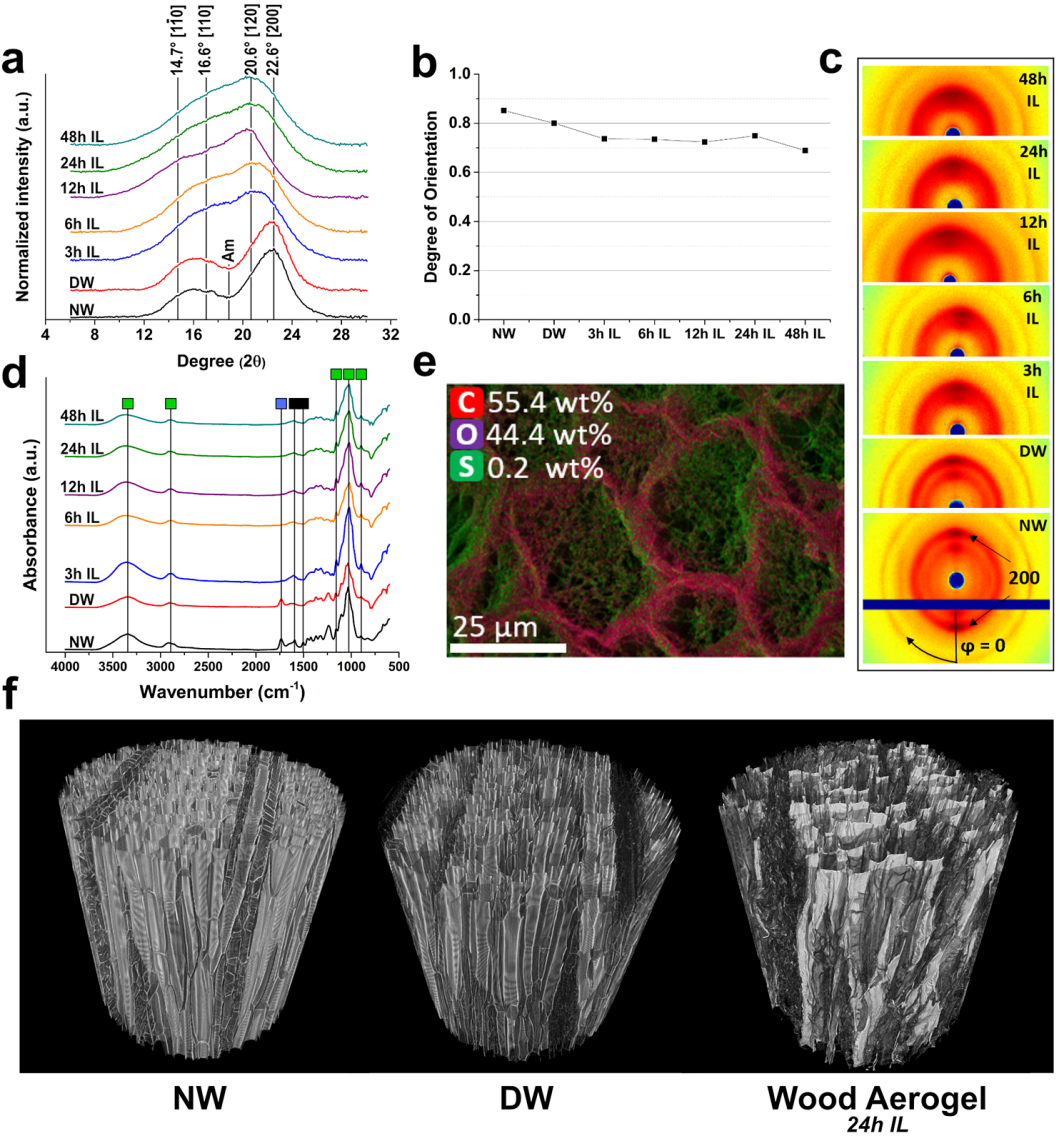
(a) WAXS spectra for NW, DW, and all IL treated wood (3 h, 6 h, 12 h, 24 h, 48 h). The cellulose I crystalline planes and the amorphous contribution are displayed with lines. (b) Degree of orientation of aligned cellulose nanofibrils in NW, DW, and all IL treated wood (3 h, 6 h, 12 h, 24 h, 48 h). (c) WAXS images of NW, DW, and all IL treated wood (3 h, 6 h, 12 h, 24 h, 48 h). (d) FT-IR spectra for all specimens including NW, DW, and all IL treated wood (3 h, 6 h, 12 h, 24 h, 48 h); main signal from lignin (black), hemicelluloses (blue) and cellulose (green) are shown. (e) EDX image of a 24 h wood aerogel; elements are green for sulfur, red for carbon, and blue for oxygen. (f) μCT images of NW, DW, and 24 h IL-treated wood aerogel.

Wide-angle X-ray scattering (WAXS) was conducted to provide the wood cellulose crystalline structure and orientation change. Both NW and DW show distinct cellulose I_β_ peaks (14.7°, 16.6°, 20.6°, and 22.6° in Figure 4a). A significant change of the cellulose crystalline structure was observed for all wood aerogels as amorphous cellulose halo increased with reaction time. The amorphous contribution dominated the spectrum even at 3 h of IL treatment. Thereafter, a further shift toward amorphous cellulose was observed, where the slope over (1–10) and (110) became steeper. The remaining shoulder from 14° to 17° is likely remnants of (1–10) and (110) crystalline planes of cellulose I.^21^ The crystalline structure change supports our hypothesis of cell wall dissolution and regeneration. Based on these observations, the DMSO/IL is believed to readily permeate the entire cell wall, rapidly interacting with the cell wall biopolymers (Figure 4a and Figure 3b). Thereafter, the dissolved cell wall molecules (mainly cellulose and hemicellulose) diffused from the compact wood cell wall to the liquid bulk in central lumen space of fibers, which is the limiting factor for nanofibril network formation inside the lumen (Figure 3b–f and Figure 4a). This provides us with temporal control of the network formation. The retention of nanofibrils in the lumen space, is due to entrapment of the dissolute, which is unable to escape the compact wood cell walls within the time-scale.

Despite a substantial change in crystallinity, noncrystalline ordering is apparent. The nanofibril orientations in NW, DW, and wood aerogels are presented in Figure 4b. The orientation degree was determined from the WAXS images in Figure 4c, where NW and DW showed high orientation degrees of 0.85 and 0.8, respectively. For wood aerogels, the nanofibril network fraction in the lumen is close to randomly oriented in space, leading to decreased average cellulose orientation. Initially (3 h), a rapid decrease in orientation to 0.74 was observed, which remained stable until 24 h. After 48 h, the orientation decreased to 0.69. The present orientation is notably high compared to other cellulose-based aerogels and anisotropic aerogels, even comparable with unidirectional freeze-casted CNF-foams.^22,23^ The possible reason for such high orientation is the inherent orientation of the microfibrils remained in the cell wall (Figure 1a).

Fourier transform infrared spectroscopy (FT-IR) was performed to investigate chemical changes during the processing (Figure 4d). Delignification was effective as prominent lignin peaks (1596 and 1505 cm^–1^)^24^ receded for DW. The following IL treatment did not affect the molecular cellulose structure, as the intrinsic bonds remained relatively unaltered. Peaks corresponding to C–O–C (1150 cm^–1^),^25^ polysaccharide rings (1034 cm^–1^), and C–O–C of β-glycosidic bonds (893 cm^–1^) remained fairly unchanged.^26^ Signals for −OH (3400 cm^–1^) and C–H (2900 cm^–1^) also remained stable. C=O at 1732 cm^–1^ (representing acetyl groups) vanish and the drastic decrease of CH signal at 1112 cm^–1^ could be attributed to hemicelluloses structure change such as deacetylation or removal.^27^ Carbohydrate analysis shows a slight loss of xylan during the first 3 h of IL treatment (Figure S14), which is in line with the FT-IR data. Energy dispersive X-ray spectroscopy (EDX) (Figure 4e) revealed trace amounts of sulfur solely associating with nanofibrils in the lumen, indicating the important role of DMSO in this process. X-ray microtomography (μCT) analysis (Figure 4f) further unveiled the structural changes. NW and DW showed long fibers with empty lumen, where cell walls of DW indicated loss of intensity, due to lignin removal. The aerogel also exhibited long intact fibers, but with clear signs of networks within the lumen. It should be noted that only clusters of nanofibrils in the lumen can be detected due to μCT resolution limitation (∼700 nm). Furthermore, ray cells of the aerogel are inferred to readily dissolve, as networks appear to occupy these regions. Microtomography videos of NW, DW, and 24 h aerogels are presented in SI Videos 1, 2, and 3, respectively.

**Figure 5:**
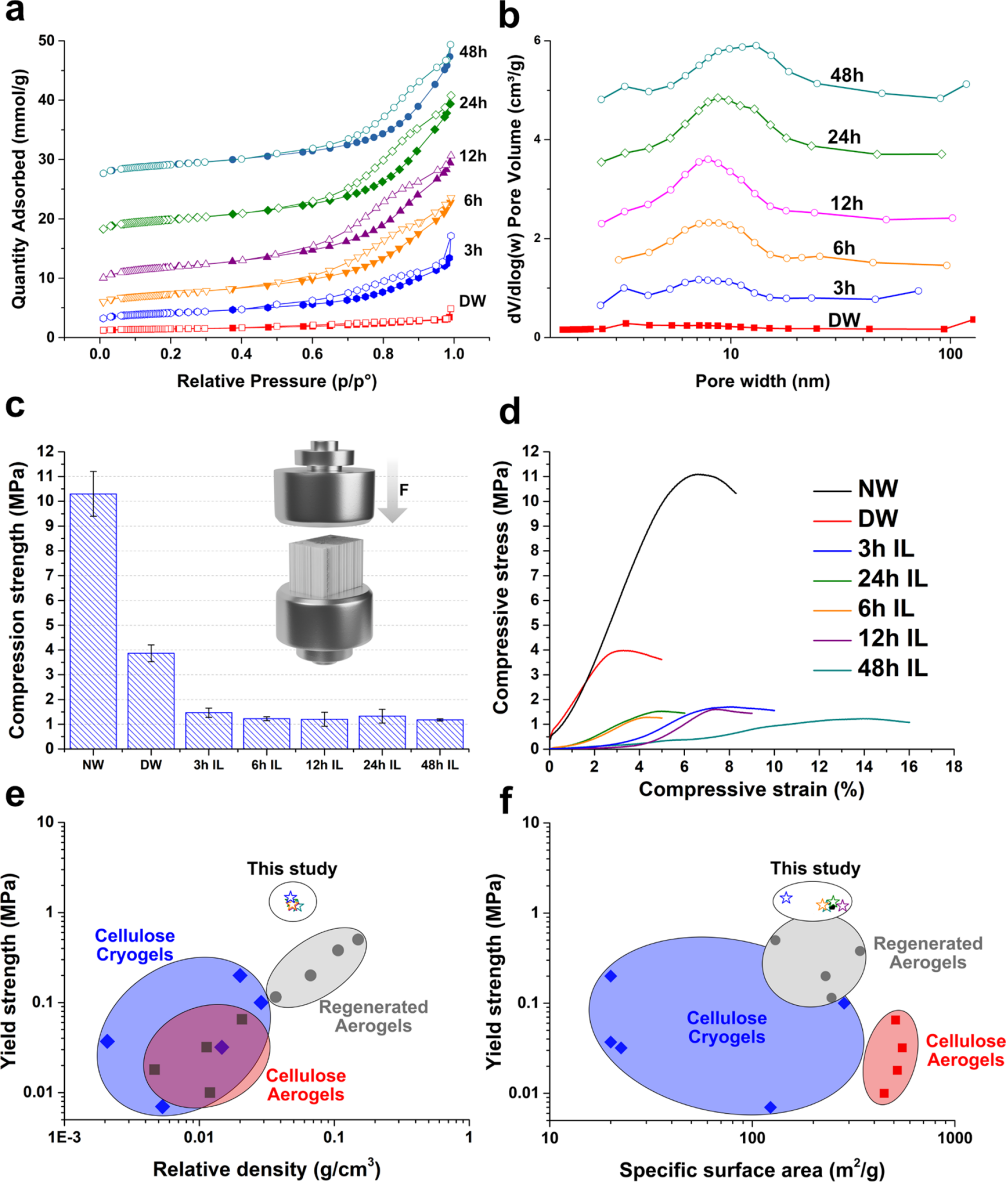
(a) Nitrogen adsorption and desorption isotherms of NW, DW, and aerogels from all consecutive IL treatment times. Filled symbols represent adsorption, hollow symbols desorption. (b) BJH Pore-size distribution of all treated wood materials. (c) Histogram of average compression strengths, (d) Typical stress–strain curves of NW, DW, and aerogels. (e) Yield strength against relative density and (f) yield strength against SSA for cellulose cryogels (freeze-dried),^28−32^ cellulose aerogels (CPD),^7,33^ regenerated aerogels (CPD)^34−36^ and our wood aerogels (CPD).

The present aerogels with nanofibrils network filling the lumina showed greatly improved SSA. DW showed a BET SSA of 39 m^2^/g higher than NW (1 m^2^/g). This is due to nano porosity generation in the cell wall after delignification. Figure 5a shows the nitrogen sorption isotherms and Figure 5b exhibits pore-size distributions (PSD). After IL treatment, all samples obtain type-IV isotherms. Large hysteresis loops in desorption are related to capillary condensation in open-ended mesopores (Figure 3a–f). An increased mesoporosity was observed (Figure 5b) due to the cell wall dissolution and the developed cellulose networks in the lumen (Figure 3b). SSA was increased with a maximum value of 279 m^2^/g (12 h IL treatment). To the best of our knowledge, this value is the highest SSA for a top-down wood cellulosic aerogel.^14,10,37,38^ Further increase of the treatment time, the SSA incrementally decreased to 233 m^2^/g (48 h IL treatment). This is explained as enlargement of mesopores in the lumen cellulosic nanofibril network (Figure 3d–f).

The mechanical properties are presented in Figure 5c,d, with yield strength (σ_y_) and Young′s modulus (*E*) compiled in Table 1. NW and DW showed initial linear stress–strain regions occurring at 0–5% and 0–2%, respectively. The high Young′s modulus and high yield strength of NW (*E* = 222 MPa, σ_y_ = 10 MPa) is attributed to the well-structured honeycomb cells with cell-walls of close to axially oriented cellulose nanofibrils. Upon delignification, much of the stiffness and strength was lost (*E* = 50 MPa, σ_*y*_ = 4 MPa) as the lignin component was removed. The aerogels showed different behaviors, with an initial region of low stress, followed by a linear region. This initial region is attributed to an artifact from imperfect specimen geometry at edge surfaces. The highest strength is for 3 h IL treatment (1.47 MPa), followed by lower, but fairly constant, strength with increasing treatment time until 48 h (1.18 MPa). The reduced stiffness (3 h treatment, *E* = 31 MPa) compared with DW could be related to decreased fraction of “preserved” cell wall (see upper row of Figure 3). Nanofibrils within lumen appear to be beneficial to longitudinal compressive yield strength by modification of the cell wall buckling mechanism for wood.^39^ Thereafter, even with a less “preserved” cell wall, the Young′s modulus of aerogels with 6 h treatment (*E* = 35 MPa) and 12 h (*E* = 45 MPa) treatment show an increase. Further increase in treatment time, the mechanical properties decreased as cell wall “thinning” dominated, leading to Young′s modulus decrease to a minimum value of 14 MPa (48 h). Supporting tensile tests in the weak direction of wood, transversal, can be seen in SI Figure S15. Transversal tensile test followed the same trend as compression tests.

**Table 1:** Nitrogen Adsorption-Desorption and Mechanical Properties of All Samples

	BET SSA (m^2^/g)	average pore diameter (nm)	yield strength σ_y_ (MPa)	Young’s modulus E (MPa)
NW	1	152	10.3 ± 0.90	222 ± 12
DW	40.5 ± 1.1	12.5 ± 0.46	3.86 ± 0.34	50 ± 5
3 h IL	147.1 ± 0.4	13.2 ± 0.51	1.47 ± 0.19	31 ± 9
6 h IL	222.0 ± 1.1	11.7 ± 0.04	1.23 ± 0.08	35 ± 5
12 h IL	278.7 ± 1.0	10.9 ± 0.06	1.20 ± 0.28	45 ± 7
24 h IL	250.9 ± 2.1	13.3 ± 0.21	1.33 ± 0.28	33 ± 9
48 h IL	233.0 ± 2.0	13.3 ± 0.40	1.18 ± 0.04	14 ± 5

The partly preserved cellulose orientation of the wood aerogel provides alignment of stiff fibrils, resulting in strength values impossible to reach with bottom-up cellulose aerogels,^7,29−36,40^ including anisotropic aerogel synthesis via directional freeze-casting.^41^Figure 5e shows yield strength versus relative density of three types of cellulose-based aerogels prepared from (a) nanocellulose cryogels (nanocellulose substrates via cryogenic freezing and freeze-drying), (b) nanocellulose aerogels, and (c) regenerated cellulose aerogels. The wood-based aerogels in this work show higher yield strength compared with regenerated cellulose with the same or even higher density. This is mainly due to the high degree of alignment preserved from the native wood. It should be noted that the combination of high SSA and mechanical strength is ascribing to this unique dual mesoporous structure (Figure 5f).

**Figure 6:**
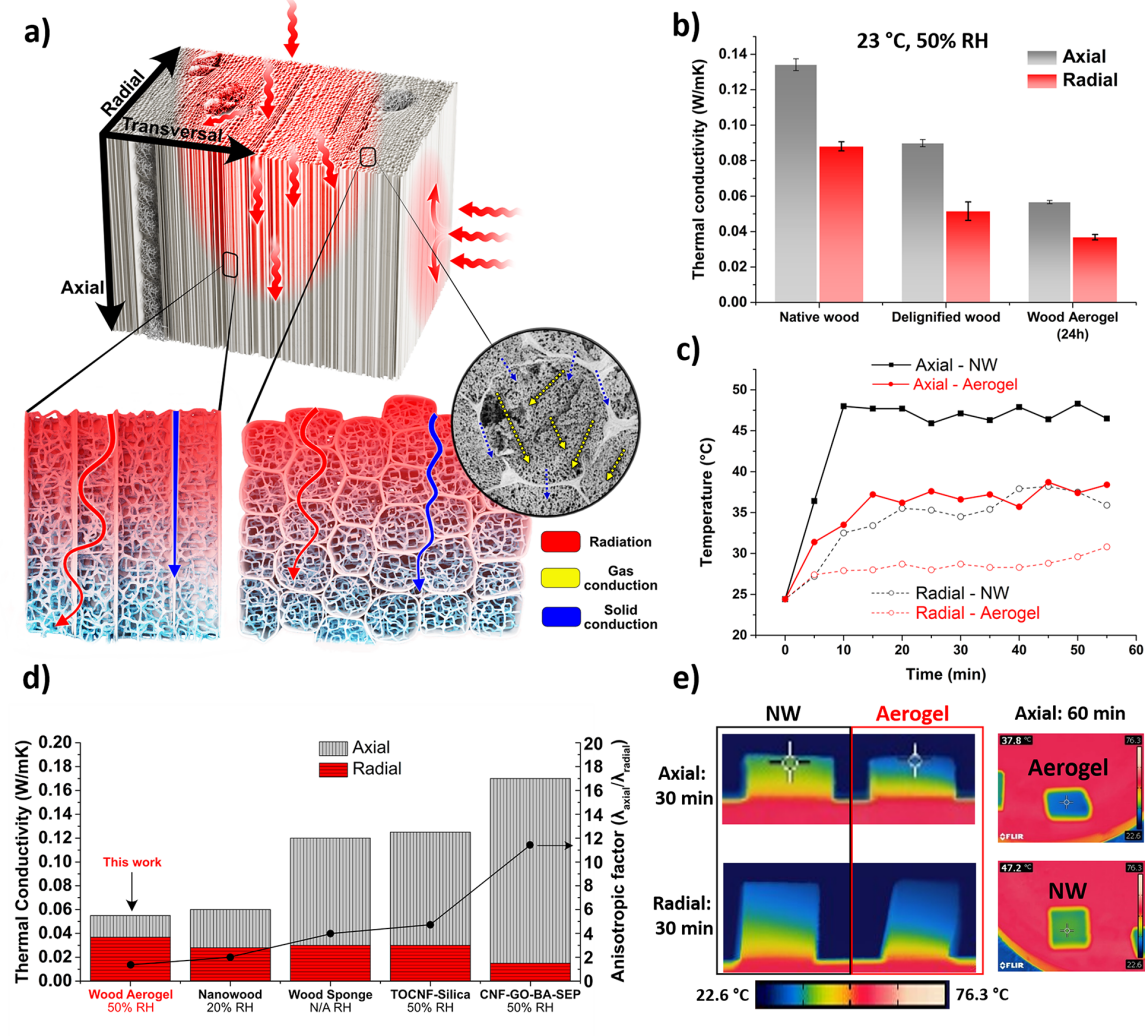
(a) Illustration of the thermal conduction in the wood aerogel. Lower illustration represents heat flow within the aerogels. Inset SEM image represents the thermal conduction in a 24 h treated wood aerogel. (b) Thermal conductivity of NW, DW, and 24 h IL treated wood aerogel from radial and axial direction. (c) IR-camera measured temperatures over time comparing NW to 24 h IL treated wood aerogel placed on a hot plate of 70 °C. (d) Thermal conductivity in this work compared to anisotropic super thermal insulators.^5,10,42,43^ (e) IR-images of NW and 24 h IL treated aerogel. Right hand-side display the temperature difference in axial-direction taken from above when laying on the hot plate. All tested samples (NW, DW, and aerogels) were obtained via freeze-drying.

The dual mesoporous structure makes the aerogel a high-performing thermal insulator. Figure 6a shows the thermal conduction in the wood aerogel and relative setup is in SI Figure S16. Apparent anisotropic thermal conductivity was observed for NW and DW (Figure 6b). The radial thermal conductivity was 0.088 and 0.051 W/mK for NW and DW, while axial conductivities were 0.134 and 0.090 W/mK, respectively. The lower values in DW are due to lower volume fraction (ρ*/ρ_s_ = 0.05) compared to NW (ρ*/ρ_s_ = 0.07), leading to decreased solid conduction in the cell wall. Cellulosic nanofibril network formation in the wood aerogel showed strong effects on the thermal conductivity. With a similar volume fraction of solids to that of DW (ρ*/ρ_s_ = 0.05), a value of 0.037 W/mK was observed in the radial direction and 0.057 W/mK was obtained in the axial for the wood aerogel. This is the lowest axial value of any anisotropic wood aerogels,^10,42^ and even lower compared to anisotropic CNF-based aerogels and foams,^5,43^ see Figure 6d. The radial thermal conductivity is comparable with CNF metal–organic framework hybrid (45 W/mK),^44^ pure CNF (53 W/mK)^44^ and CNF-polyoxamer foam (60 W/mK)^6^ at the same humidity (50% RH). Nanowood^42^ and wood-sponge^10^ (Figure 6d) show slightly lower radial conductivity, but note the lower RH of 20%. Contributing mechanisms to the low thermal conductivity of wood aerogel are illustrated in Figure 6a. The thermal conductivity is expressed as a sum of convection, radiation of photons, gas, and solid conduction. Compared with DW, the wood aerogel shows lower gas diffusion ascribing to the nanofibril network in the lumen (Figure 6a). The nanoporosity of the aerogel (average pore-size ∼20 nm) is decreasing the average mean free path of air (∼50 nm),^11^ also called the Knudsen effect, leading to reduction in thermal conductivity.^11^ Consequently, the contribution from solid conduction is reduced due to the great amount of gas/solid interfaces. In addition, natural convection does not occur in nanosized pores^45^ and radiation is minor, as absorption and scattering by cell walls and nanofibrils reduce this contribution.^46^ Pore-size distribution of the freeze-dried 24 h IL treated wood aerogel used for thermal properties measurements is seen in SI Figure S17. The λ_axial_/λ_radial_ value, which represents the thermal insulation anisotropy, is 1.54 (1 represents isotropic), indicating nearly isotropic thermal insulation property. The value is the lowest for the current reported anisotropic aerogel, see Figure 6d.^6,10,42,43,47^ The reason is that cellulosic nanofibrils network in the lumen is isotropic and dominates the thermal conductivity in both directions.

To investigate the thermal insulation function of the wood aerogel, upper surfaces temperature changes over time of samples placed on hot plate of 70 °C were recorded using IR thermal camera (Figure 6c). All IR-images are presented in SI Figure S18. With the axial direction parallel to heat flow, the temperature stabilized after 10 min at ∼47 °C for NW, while for wood aerogel the stabilization time is longer (15 min) and stabilized temperature is much lower (∼36 °C). Even after 60 min, the difference between the two materials remained, indicating the superior thermal insulation of wood aerogel. In the radial direction, both specimens showed stable temperature after 20 min and ∼26 °C for the aerogel (∼35 °C for NW). Figure 6e shows the temperature gradient of NW and wood aerogel after heating for 30 and 60 min. The suppressed axial conduction in the aerogels compared with NW is particularly obvious, although the difference in temperature profile between NW and aerogel in the radial direction is already apparent. Thermal diffusivity and specific heat capacity of treated samples are seen in SI Table S2 and Figure S19, respectively. The combined high mechanical property and low thermal conductivities in both axial and radial directions make the wood aerogel an attractive high performance thermal insulator.

## Conclusion

A strong and high-performance thermal insulating wood aerogel with cellulosic nanofibril networks occupying the lumen space is achieved through wood cell wall dissolution and controlled precipitation in the lumen space, using a mixture of DMSO and the IL [MTBD]^+^[MMP]^−^. The cellulosic nanofibril network formation was controllable through tailoring the cell wall dissolution and dissolute diffusion. High degree of orientation is obtained mainly ascribing to the inherent orientation of the microfibrils remaining in the cell wall. With this unique structure, a combination of high SSA up to 280 m^2^/g and high yield strengths (>1.2 MPa) was realized. Low thermal conductivities in both radial direction (0.037 W/mK) and axial direction (0.057 W/mK) was realized, which is rare in the literature. The high strength combined with excellent thermal insulating properties, sets the aerogel apart from most cellulose-based thermal insulators. The facile and potentially scalable production method, the controllability on the aerogel nanostructure, and excellent properties reported contribute greatly toward sustainable high performing aerogels design for advanced applications such as super thermal insulation.

## Methods

### Materials

Balsa (Ochroma pyramidale) with a density of 111 ± 10 kg/m^3^ was bought from Material AB, Sweden. The wood was cut to the dimensions 15 × 15 × 10 mm^3^ (tangential × radial × axial). Sodium chlorite, acetic acid and sodium acetate were bought from Sigma-Aldrich, Sweden. DMSO was purchased from Sigma-Aldrich, Sweden. 1,1,3,3-tetramethylguanidine (+98%) was purchased from fluorochem and freshly distilled prior to use. MTBD and MTBN were obtained from Liuotin group OY. Trimethylphosphate and dimethyl methylphosphonate (DMMP) (+99%) were purchased from Merck or ABCR GmbH.

### Delignification of Balsa Wood

Balsa wood was introduced to the delignification chemicals, 1 wt % sodium chlorite in an acetate buffer (pH 4.6), wherein delignification transpired for 12 h at 80 °C. The buffer solution was renewed after 6 h. Subsequently, the delignified wood was thoroughly rinsed with Milli-Q water.

### Preparation of Aerogels

Delignified wood was immersed in DMSO, the solvent exchange was repeated three times to ensure no water in the structure. Thereafter, the specimens were introduced to an [MTBD][MMP]:DMSO 20:80 wt % electrolyte, wherein they were treated for a designated amount of time (3 h, 6 h, 12 h, 24 h, or 48 h) at 65 °C. The gel-substrates were regenerated through immersion of Milli-Q water. The rinsed samples were then dried by rapid freezing using liquid nitrogen (−196 °C) by placing the samples on an aluminum dish floating on the LN_2_ for about 30 s, followed by freeze-drying (−90 °C), for at least 2 days. Samples used for nitrogen physisorption were dried using critical point drying for 20 min. These samples were first solvent exchanged from water to ethanol, followed by CPD in an Autosamdri-815.

### Ionic Liquid Preparation and Characterization

Distilled MTBD (613 g, 3.6 mol, 1.2 equiv), previously stored under Argon, was poured in a 2 L two-necked round-bottom flask with dimethyl methylphosphonate (DMMP) (384 g, 3 mol, 1.0 equiv). Then, the flask was evacuated and backfilled with Argon. Subsequently, the mixture was allowed to stand at room temperature for 10 min to gently dissipate the mild exothermic reaction observed and heated up at 110 °C with vigorous stirring. Once the reaction was completed (ca. 18 h, as it was observed by NMR analysis of a reaction aliquot, see SI), the mixture was allowed to cool down at room temperature. The titled ionic liquid was obtained as a highly pure dark yellow liquid that did not require further purification.

The ionic liquids thermal stability from 30 to 500 °C was studied by thermogravimetric analysis (TGA, Mettler Toledo TGA/DSC1, Switzerland) with a heating rate of 10 °C min^–1^ (N_2_ atmosphere, 50 mL min^–1^). Differential scanning calorimetry (DSC) curves was obtained with a Mettler Toledo DSC1 instrument (Switzerland), using a heating rate of 10 °C min^–1^ between 30 and 500 °C under a nitrogen flow of 50 mL min^–1^. Temporal degradation of the IL was studied using a Proton Nuclear Magnetic Resonance (1H NMR), performed in a Bruker AM 400 instrument at 400 MHz. The IL was investigated using deuterated dimethyl sulfoxide. Peak analysis was performed with the software MestReNova 9.0.

### Aerogel Characterization

Morphology of DW and IL treated wood aerogels were examined with a field emission scanning electron microscope (SEM, Hitachi S-4800, Japan). Prior to SEM analysis, all specimens were sputtered with a platinum/palladium layer of about 3 nm. Sputtering was performed using a Cressington 208HR, UK, for 20 s. To assess the elemental composition of the specimen, energy dispersive spectroscopy (Oxford Instruments, X-MAX N 80, UK) was used. The EDS was an extension on the FE-SEM, in which an accelerating voltage of 10 kV and a working distance of 15 mm was utilized. The composition was gathered using a mapping technique.

Density measurements were performed by oven drying (105 °C) the specimen overnight. Before weighing, the specimens were placed under vacuum for 30 min. From the dry weight and caliper (Mitutoyo, digital Caliper) dimension measurements the porosity was assessed. The porosity was determined with eq 1. Solid density of NW and DW was assigned 1500 kg/m^3^.^39^
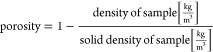
1

Small angle X-ray measurements were performed using a Mat:Nordic from SAXSLAB with a Rigaku 003+ high brilliance microfocus CuK alpha radiation source and a Pilatus 300 K detector. The measured q-range was 0.007–2.5 Å^–1^ with the exposure time of 10 min. Thin slices were cut through the longitudinal direction of each specimen, thereafter mounted onto a metal holder. To attain the 2D diffraction patterns, the specimens were positioned perpendicularly to the incident beam. The resulting diffractograms were imported to ImageJ in which the nanofibril orientation of cellulose was obtained by azimuthal integration along the Debye–Scherrer ring of the intense crystal plane (200). The intensity profiles (2 per specimen) were Gaussian fitted to observe the full width at half-maximum (fwhm), which could be used in eq 2 to obtain the degree of orientation. The attained orientation value (*f*) can maximally have a value of 1, representing perfect anisotropy and minimally 0, representing complete isotropy.
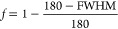
2

Attenuated total reflectance Fourier transform infrared (ATR-FTIR) was performed utilizing a PerkinElmer Spectrum 100 FTIR spectrometer equipped with an ATR unit (Graseby Specac LTD, England). The spectra were obtained using a resolution of 4 cm^–1^ over the range of 4000–600 cm^–1^ using eight scans.

Specific surface area and pore size distribution were measured by nitrogen physisorption. CPD dried samples each of about 0.3 g were outgassed at 80 °C for 1 day, followed by the physisorption analysis. The analysis was carried out in a Micromeritics ASAP 2020 between the relative pressures 0.05–1.0 *P*/P_o_ under liquid nitrogen conditions of −196 °C, wherein the BET surface area was determined between the relative pressures 0.05–0.25. The BET SSA was evaluated using nitrogen adsorption–desorption. The isotherms were assessed with the Brunauer-Emmet-Teller (BET) theory for SSA and the pore-size distribution (PSD) and pore-volume was assessed with the Barret-Joyner-Halenda (BJH) model.^48^

Carbohydrate and lignin content of each specimen was measured by grinding the specimen in a Wiley mill and subsequently hydrolyzing the ground material in sulphuric acid (73%). The carbohydrate constituents were determined through introduction of the hydrolyzed substance to a Dionex ICS-300 ion chromatography system (Thermo Fisher Scientific Inc.). The lignin portion was assessed with the standard method: TAPPI T 222 om-2, also called Klason lignin.

X-ray tomography was performed using a Zeiss XRadia Versa XRM520. The X-ray tube voltage and power were set to 80 kV and 7 W, respectively. The samples were placed 8.03 mm from the source and at 7.15 mm from the detector. 3201 radiographs were acquired over 360° with an exposure time of 3 s per projection using 20× optical magnification. The tomographic reconstruction provided a cylindrical image of the internal central section of the sample with both diameter and height of about 2000 voxels, where the voxels are cubic with side lengths of 354 nm, giving a field of view of about 700 μm in diameter and in height. The estimated 3D spatial resolution, based on the instrument calibration is about 700 nm. The tomographic reconstruction was performed using the Zeiss reconstruct software with a correction for the center of rotation and output as 16-bit tiff slices.

Mechanical properties were evaluated via longitudinal compression, utilizing a Instron 5966 with a load cell of 10 kN at a strain rate of 10%/min. All mechanical compressions were performed in a conditioning room of 50% relative humidity and 23 °C. Prior to mechanical testing, all samples were conditioned for at least 3 days. All samples had an approximate dimension of 10 × 15 × 15 mm^3^ (longitudinal × radial × tangential). Mechanical compression strength was evaluated by observing the stress at structural collapse, also called the yield point. At this point, a plastic deformation initiates, in which the material no longer can recover elastically. The yield point was established at the intersection of the tangent line from the linear elastic region and the tangent line from the plateau region. From the stress–strain curve, Young′s modulus could be obtained from the slope of the elastic linear region. Transversal tensile tests were performed using a Instron 5944 with a 500 N load cell at a strain rate of 3 mm/min. All samples had an approximate dimension of 10 × 3 × 15 (longitudinal × radial × tangential).

Thermal conductivity of freeze-dried samples (NW, DW and wood aerogel 24h) were analyzed using a TPS 2500 S thermal Constants analyzer (Hot disk, Sweden) in anisotropic mode. The measurements were performed at a heating of 20 mW, the measurement time was 5 s for each respective sample. The measurements were performed in an ambient environment of 23 °C and 50% RH. Each sample measurement consisted of two identical samples (25 × 25 × 25 mm^3^) sandwiched between the transient plane sensor (3.2 mm radius). Each sample was measured 5 times with an interval of 10 min between measurements. All samples were conditioned in 23 °C and 50% RH for at least 2 days prior to analysis. The specific heat capacity (*C*_p_) needed for anisotropic TPS measurements was measured with DSC (Mettler Toledo DSC1, Switzerland) using a sapphire standard. To ensure *C*_p_ of dry specimen the samples were predried at 105 °C in an oven. In addition, a drying protocol was performed in the DSC for 30 min at 105 °C followed by a dynamic measurement between −20 and 50 °C at 10 °C/min under N_2_ atmosphere. Infrared thermographic images were obtained by placing native wood and wood aerogels on a hot plate with stable temperature of 70 °C. Thermographic images were taken every 5 min using a FLIR E60 with resolution of 320 × 240 pixels.

## Supporting Information Available

Preparation of different ionic liquids; screening of ionic liquids with enocell pulp; ^1^H NMR analysis of [MTBD]^+^[MMP]^−^; ^31^P NMR analysis of [MTBD]^+^[MMP]^−^; ^13^C NMR analysis of [MTBD]^+^[MMP]^−^; NMR and ATR-IR analysis of [MTBD]^+^[MMP]^−^; ^31^P NMR, chemical stability of [MTBD]^+^[MMP]^−^ of 1.0:1.0; ^31^P NMR, chemical stability of [MTBD]^+^[MMP]^−^ of 1.2:1.0; thermal analysis of [MTBD]^+^[MMP]^−^; low magnification SEM images in radial, transversal and longitudinal direction of aerogel; high magnification of lumen fibril network structure; low magnification SEM images of all treated samples; visual dimensional changes of ionic liquid treated samples; upscaling and different geometries; detailed carbohydrate data of all samples; transversal tensile tests of all specimens; hot disk thermal conductivity setup; pore-size distribution of freeze-dried 24 h IL wood aerogel; IR-thermographic images; specific heat capacity of NW, DW and aerogel; density, weight loss and dimensional changes of all samples; thermal diffusivity of the tested samples (PDF)
Tomography video of NW (MPG)
Tomography video of DW (MPG)
tomography video of 24 h IL (MPG)

## Supplementary Material

am2c04584_si_001.pdf

am2c04584_si_002.mpg

am2c04584_si_003.mpg

am2c04584_si_004.mpg
