# Supplementary material for: Nanostructurally Controllable Strong Wood Aerogel toward Efficient Thermal Insulation
Source: ACS Appl Mater Interfaces. 2022 May 5;14(21):24697–707. doi: 10.1021/acsami.2c04584 (PMC9164199; doi:10.1021/acsami.2c04584)
Supplement: Supplementary file 1 — am2c04584_si_001.pdf [file am2c04584_si_001.pdf]

# Supporting Information:

# Nanostructurally Controllable Strong Wood Aerogel towards Efficient Thermal Insulation

*Jonas Garemark[1], Jesus E. Perea-Buceta[2], Daniel Rico del Cerro[2], Stephen Hall[3], Barbara Berke[4], Ilkka Kilpeläinen[2], Lars A. Berglund[1], Yuanyuan Li[1]**

[1]Wallenberg Wood Science Center, Department of Fiber and Polymer Technology, KTH Royal Institute of Technology, SE-10044 Stockholm, Sweden

[2] Department of Chemistry, University of Helsinki, A.I. Virtasen aukio 1, 00560 Helsinki, Finland

[3]Lund University, Division of Solid Mechanics, SE-221 00 Lund, Sweden

[4]Department of Physics, Chalmers University of Technology, 412 96 Gothenburg, Sweden

*Corresponding author email: yua@kth.se

Table of contents:


**1. Chemistry**

**1.1 Materials and methods for ionic liquids preparation and characterization**

**1.2 Ionic liquids (ILs)**

1.2.1. IL design

1.2.2. Screening of phosphorous-based guanidine type IL

1.2.3. Synthesis and characterization of $[MTBD]^+[MMP]^-$

1.2.4. Stability of $[MTBD]^+[MMP]^-$

**2. Aerogel supplementary results**

**Supplementary figures**

Figure S1. Guanidinium-based and phosphorous-based ionic liquids studied

Figure S2. Screening of Enocell pulp (3 wt.%) treatments in different IL:DMSO (80:20) electrolytes

Figure S3. $^1$H NMR analysis of MTBD and $[MTBD]^+[MMP]^-$

Figure S3. $^{31}$P NMR analysis of $[MTBD]^+[MMP]^-$

Figure S4. $^{13}$C NMR analysis of $[MTBD]^+[MMP]^-$

Figure S5. NMR and ATR-IR analysis of $[MTBD]^+[MMP]^-$

Figure S6. $^{31}$P NMR, chemical stability analysis of $[MTBD]^+[MMP]^-$ of molar ratio 1.0:1.0

Figure S7. $^{31}$P NMR, chemical stability analysis of $[MTBD]^+[MMP]^-$ of molar ratio 1.2:1.0

Figure S8. Thermal analysis of $[MTBD]^+[MMP]^-$

Figure S9. Low magnification SEM images in radial, transversal and longitudinal direction of aerogel

Figure S10. High magnification of lumen fibril network structure

Figure S11. Low magnification SEM images all treated samples

Figure S12. Visual dimensional changes of ionic liquid treated samples

Figure S13. Upscaling of wood aerogels.

Figure S14. Detailed carbohydrate data of all samples

Figure S15. Transversal tensile tests of all samples

Figure S16. Hot disk thermal conductivity set-up

Figure S17. Pore-size distribution of freeze-dried 24h IL wood aerogel.
Figure S18. IR-thermographic images
Figure S19. Specific heat capacity of NW, DW and aerogel

### Supplementary Tables

Table S1. Density, weight loss and dimensional changes of all samples
Table S2. Thermal diffusivity of the tested samples

### 3. References

## 1. Chemistry

### 1.1 Materials and methods for ionic liquids preparation and characterization

Unless stated otherwise, all the chemicals and deuterated solvents were purchased from commercial sources at the highest degree of purity available and used without further purification. 1,1,3,3-Tetramethylguanidine (+98%) was purchased from Fluorochem and freshly distilled prior use. MTBD and MTBN were obtained from Liuotin group OY. Trimethylphosphate and Dimethyl methylphosphonate (DMMP) (+99%) were purchased from Merck or ABCR GmbH. The syntheses of ionic liquids were conducted under an **argon atmosphere** using conventional Schlenk techniques on a dual-manifold gas-inlet/vacuum line. All glassware was flame-dried prior to use. HPLC-quality grade reaction solvents were dried by conventional methods,. **The solvents** required to conduct the rest of synthetic operations were purchased at HPLC-quality grade (99.9% Honeywell, or Fischer Scientific) and used as received.

**All nuclear magnetic resonance (NMR) experiments** ($^{1}H$, $^{13}C$, $^{19}F$) were performed on Bruker Avance NEO spectrometers operating with the frequency, deuterated solvent and at the temperature indicated in parentheses. All chemical shifts values (δ) are reported in parts per million (ppm) downfield in relation to tetramethylsilane using the residual undeuterated solvent signal as secondary internal standard ($CHCl_3$ in $CDCl_3$; $\delta_H$ = 7.26 ppm and $\delta_C$ = 77.16 ppm), ($CHDCl_2$ in $CD_2Cl_2$; $\delta_H$ = 5.32 ppm and $\delta_C$ = 53.8 ppm), ($C_6HD_5$ in $C_6D_6$; $\delta_H$ = 7.16 ppm and $\delta_C$ = 128.1 ppm). The resonances on the $^{19}F$-NMR spectra are reported in parts per million (ppm) downfield of $CFCl_3$, using hexafluorobenzene ($C_6F_6$ at –164.9

ppm) as internal standard. Unless otherwise noted, all coupling constants (*J*) are quoted to the nearest 0.1 Hz with the involved nuclei subscripted, and the resonances are noted as follows: $^1$H: δ chemical shift in ppm (number of protons, multiplicity, *J* value(s), assignment). $^{13}$C: δ chemical shift in ppm (multiplicity [if applicable], *J* value(s) [if applicable], assignment). $^{19}$F: δ chemical shift in ppm (number of fluorines, multiplicity, *J* value(s), assignment). Splitting patterns are denoted as s (singlet), d (doublet), t (triplet), q (quartet in $^1$H-NMR, quaternary in $^{13}$C-NMR), p (pentet), m (multiplet), dd (doublet of doublets), ddd (doublet of doublet of doublets), dt (doublet of triplets), td (triplet of doublets), br (broad resonance), app (apparent). Unless otherwise stated, both the resonances on the $^{13}$C and $^{19}$F-NMR spectra were proton-decoupled.

**High-resolution electrospray-ionization mass spectra** (ESI-MS) were recorded on a Bruker microTOF mass spectrometer operated in a positive or negative ion mode, using a 0.05 M solution of sodium formiate as a calibrant. Stock solutions (400-1000 ppm) were prepared of acetonitrile (99.9% Honeywell, Riedel-de Haën) and were diluted (1 ppm) with acetonitrile prior to the measurement. Only the prevalent ion peak ($HCOO^-$, $H^+$ or $Na^+$ adduct) is given for each compound.

**IR** was performed by Attenuated Total Reflect IR spectroscopy (ATR-IR) using an Alpha-P IR spectrometer from Bruker Optics. Thermogravimetric analysis (**TGA**) was used to determine the thermal stability of the ionic liquids on the range 30-500 °C using a Mettler Toledo TGA/DSC1, Switzerland device with a heating rate of 10 °C min$^{-1}$ ($N_2$ atmosphere, 50 mL min$^{-1}$). Differential scanning calorimetry (**DSC**) curves were obtained with a Mettler Toledo DSC1 instrument (Switzerland), using a heating rate of 10 °C min$^{-1}$ between 30-500 °C under a nitrogen flow of 50 mL min$^{-1}$.

## 1.2. Ionic liquids (ILs)

### 1.2.1. IL design

In contrast to conventional molecular liquids, ILs are amphiphilic in nature and behave like nano-heterogeneous media that self-assemble into polar and hydrophobic non-polar domains mainly governed by Coulombic electrostatic forces.[S1,2] This amphiphilicity perfectly matches the anisotropic character of cellulose where relatively hydrophobic sheets, paired against one another, co-exist with polar chains linked by hydrogen bonding networks.[S3] Therefore, ILs are endowed with an enormous potential to participate in key processes for aerogel formation such as the dissolution of lignocellulosic materials with unique control of the directionality of the molecular interactions involved.[S3,4] To that end, the optimum ILs feature small anions with hydrogen-bond basicity high enough – such as halides or polyatomic anions with delocalized charge – to establish strong hydrogen bond interactions with the hydroxyls in equatorial positions of the cellulosic chains.[S4,S5] Additionally, the ILs must contain small non-coordinating cations without long aliphatic chains or protic groups that obstruct cellulose dissolution by creating instead hydrogen bonds with the ILs anions. As a result, ILs cations also contribute to dissolve cellulose by establishing van der Waals and dispersion forces with the surfaces above and below the polar glucose chains, that replace the stacking hydrophobic interactions holding together the sheets in crystalline cellulose.[S3,S6]

To begin our studies, we devised that ILs obtained from trimethyl phosphate (TMP) **4** and dimethyl methylphosphonate (DMMP) **5** could be ideal model solvents to evaluate the preparation of aerogels from wood materials (Figure S1).[S7,8] Indeed, such ILs are formed by methyl transfer from those phosphorous-based reagents, and thus can be protic or aprotic depending on the choice of cationic precursor accepting that methyl group. This versatility is key to access and evaluate ILs featuring different degrees of polarity, charge delocalization, and H-bonding interactions between their cation and anion constituents, all of which may be critical factors affecting wood dissolution and aerogel formation.[S3] Furthermore, the resulting demethylated anions [DMP]⁻ and [MMP]⁻, in those ILs, possess hydrogen-bond basicity high enough to enable swift dissolution of relatively high loadings of cellulosic materials upon mild conditions.[S9-10] However, to the best of our knowledge, imidazolium scaffolds are the only cations that have been hitherto used to prepare ILs featuring [DMP]⁻ and [MMP]⁻ anions.[S7-10] To that end, the reported toxicity profile,[S11,12] relative sensitivity to moisture and tendence to react with the cellulose reducing ends of imidazolium ILs,[S13-15] prompted us to deviate our attention towards another family of *N*-based cations with delocalized charge such as guanidinium superbases **1**–**3**, that has shown intriguing applicability in the processing of wood materials.[S16-19]

In practice, the preparation of the ILs with [DMP]⁻ anions, **6** and **9**, was accomplished quantitatively by mixing the corresponding precursors in an equimolar ratio at 100-110 °C. Conversely, the only IL-based on the [MMP]⁻ anion that could be obtained in such manner was [TMG]⁺[MMP]⁻ **7**. In fact, the ILs containing bicyclic guanidinium cations and the [MMP]⁻ anion, **8** and **10**, decomposed steadily over time when they were prepared by reacting equimolar amounts of their precursors. However, we were pleased to fully stabilize both ILs, **8** and **10**, by using and slight excess of the corresponding basic guanidine precursor, **2** and **3**, (*ca.* 0.2 equiv) in their synthesis.

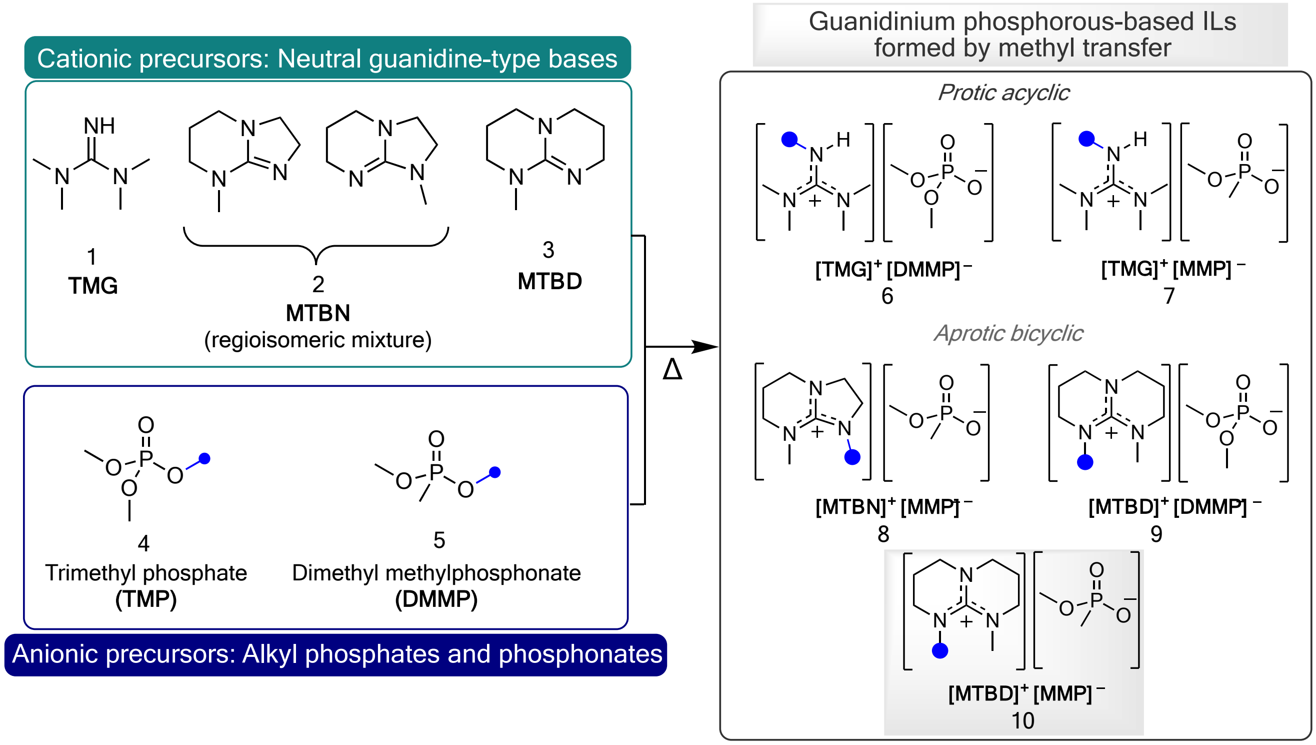


**Figure S1.** Guanidinium-based and phosphorous-based ionic liquids included in this study

### 1.2.2. Screening of phosphorous-based guanidine type IL

With the set of guanidinium phosphorous-based ILs **6**-**10** in hand, we proceeded to pre-examine their reactivity profiles towards cellulosic materials. For this task, we selected Enocell as model pulp substrate due to its low hemicellulose content. Additionally, we took into account that electrolyte solutions, arising from the combination of ILs and some polar solvents such as DMSO, are less viscous and thus diffuse better into cellulosic biopolymers, hence leading to significant rate increases of cellulose dissolution.[S20,21] Based on our previous work,[S22] we selected IL:DMSO 80:20 (wt %) as the optimum ratio. This ratio has shown to be a reasonable compromise of dissolution at relatively low viscosity. This treatment with IL:DMSO 20:80 (wt %) electrolyte led to fibrillation or in some cases partial dissolution (Figure S2).

**Neat IL experiments:**

4 ml vial was loaded with 0.03 g of Enocell (bleached hardwood pre-hydrolysis kraft pulp, Enocell Mill, Uimaharju, Stora Enso, 6.8 % xylan), and 1 g of IL (3 wt. % pulp consistency). The mixture was heated to 65 °C overnight under vigorous stirring.

**IL electrolyte experiments:**

In first place IL electrolyte solution was prepared by mixing 800 mg of DMSO-$d_6$ and 200 mg of the selected IL. In second place, a 4 ml vial was load with 0.03 g of Enocell pulp and 1 g of electrolyte solution (3 wt. % pulp consistency). The mixture was heated to 65 °C overnight under vigorous stirring. An aliquot of these mixtures was used directly for NMR analyses and another aliquot was directly analyzed by light-polarized microscopy (microscope Olympus BX51) to study the IL effect on pulp dissolution and particle morphology.

**Figure S2** shows the effect of the different IL:DMSO electrolytes in the pulp. The most promising ionic liquids for dissolution of the pulp are $[MTBD]^+[MMP]^-$ **10** and $[TMG]^+[MMP]^-$ **7**. Studies of $[MTBD]^+[MMP]^-$ by TGA showed that the IL is thermally stable up to ~260 °C. Additionally, DSC shows its melting curve around 330 °C. Therefore, we decided to select $[MTBD]^+[MMP]^-$ as the ionic liquid of choice for our studies due to its combination of properties for the treatment of lignocellulosic materials and the enhanced stability profile of MTBD as IL counter cation compared to TMG.

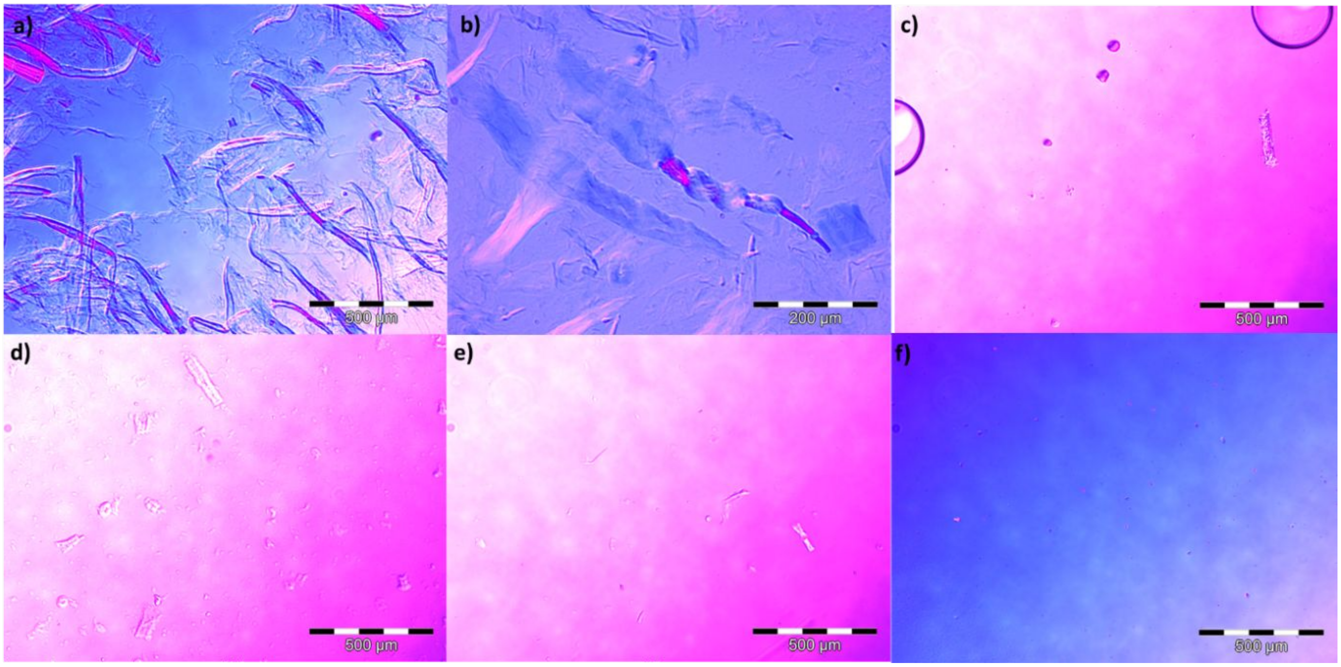


**Figure S2.** Screening of Enocell pulp (3 wt.%) treatments in different IL:DMSO (80:20) electrolytes. **a)** $[P4441]^+[DMMP]^-$:DMSO (20:80) electrolyte. Pulp without significant changes; b) $[TMG]^+[DMMP]^-$:DMSO (80:20) electrolyte. Swelling by ballooning; c) $[MTBD]^+[DMMP]^-$:DMSO (80:20) electrolyte. Almost fully dissolved; d) $[MTBD]^+[MMP]^-$ (1.25:1):DMSO (80:20) electrolyte. Fibrillation phenomena; e) $[MTBD]^+[MMP]^-$ (1:1):DMSO (80:20) electrolyte. Fibrillation phenomena too. but seems to be more effective for dissolution than d); f) $[TMG]^+[MMP]^-$:DMSO (80:20) electrolyte. Almost fully dissolved – Small sized particle.

### 1.2.3. Synthesis and characterization of $[MTBD]^+[MMP]^-$

Distilled MTBD (613 g, 3.6 mol, 1.2 equiv), previously stored under Argon, was poured in a 2 L two-necked round-bottom flask with dimethyl methylphosphonate (DMMP) (384 g, 3 mol, 1.0 equiv). Then, the flask was evacuated and backfilled with Argon. Subsequently, the mixture was allowed to stand at room temperature for 10 min to gently dissipate the mild exothermic reaction observed and warmed up at 110 °C with vigorous stirring. Once the reaction was completed (*ca.* 18 h, as it was observed by NMR analysis of a reaction aliquot), the mixture was allowed to cool down at room temperature. The titled ionic liquid was obtained as a highly pure dark yellow liquid that did not require further purification. *Note: Analogous ionic liquids (cationic bicyclic guanidinium and methyl methylphosphonate anion) prepared in a 1:1 ratio were observed to decompose over time. Using a 20% excess of the superbase was optimal to stabilize this type of ionic liquids. In practice, $[MTBD]^+[MMP]^-$ was observed to be stable in this composition at room temperature ambient conditions. However, the ionic liquid was stored and manipulated under Argon to minimize the slow hydrolysis of the guanidine base that occurs upon sustained exposure to moisture.*

**For the ionic liquid (major component):** $^{1}$**H NMR** (600 MHz, DMSO-$d_6$, 298 K): $\delta$ 3.34 (4H, app. t, $J$ = 6.3 Hz, 2 x C$\boldsymbol{H_2}$), 3.25–3.18 (7H, m, 2 x C$\boldsymbol{H_2}$ and OP(OC$\boldsymbol{H_3}$)), 2.92 (6H, s, 2 x C$\boldsymbol{H_3}$), 1.92 (4H, app. p, $J$ = 6.1 Hz, 2 x C$\boldsymbol{H_2}$), 0.78 (3H, app. d, $^{2}J_{PH}$ = 15.3 Hz, OP(C$\boldsymbol{H_3}$)); $^{13}$**C NMR** (151 MHz, DMSO-d$_6$, 298 K): $\delta$ 157.1 (q), 49.9 (d, $^{2}J_{PC}$ = 5.4 Hz, OP(O**C**H$_3$), 47.6 (2 x **C**H$_2$), 47.4 (2 x **C**H$_2$), 40.1 (2 x **C**H$_3$), 20.7 (2 x **C**H$_2$), 13.0 (d, $^{1}J_{PC}$ = 130.4 Hz, OP(**C**H$_3$)); $^{31}$**P NMR** (202 MHz, DMSO-d$_6$, 298 K): $\delta$ 16.4.

**IR (**ATR; cm$^{-1}$): 2937, 2913, 2879, 2823, 1605, 1564, 1507, 1448, 1399, 1321, 1282, 1225, 1177, 1076, 1046.

**HRMS: Cation (ESI$^+$)**: m/z calcd. for $C_9H_{18}N_3$ [M]$^+$: 168.1495, found: 168.1498; deviation: 1.556 ppm. **Anion (ESI$^-$)**: m/z calcd. for $C_2H_6O_3P$ [M]$^-$: 109.0060, found: 109.0062; deviation: 1.678 ppm.

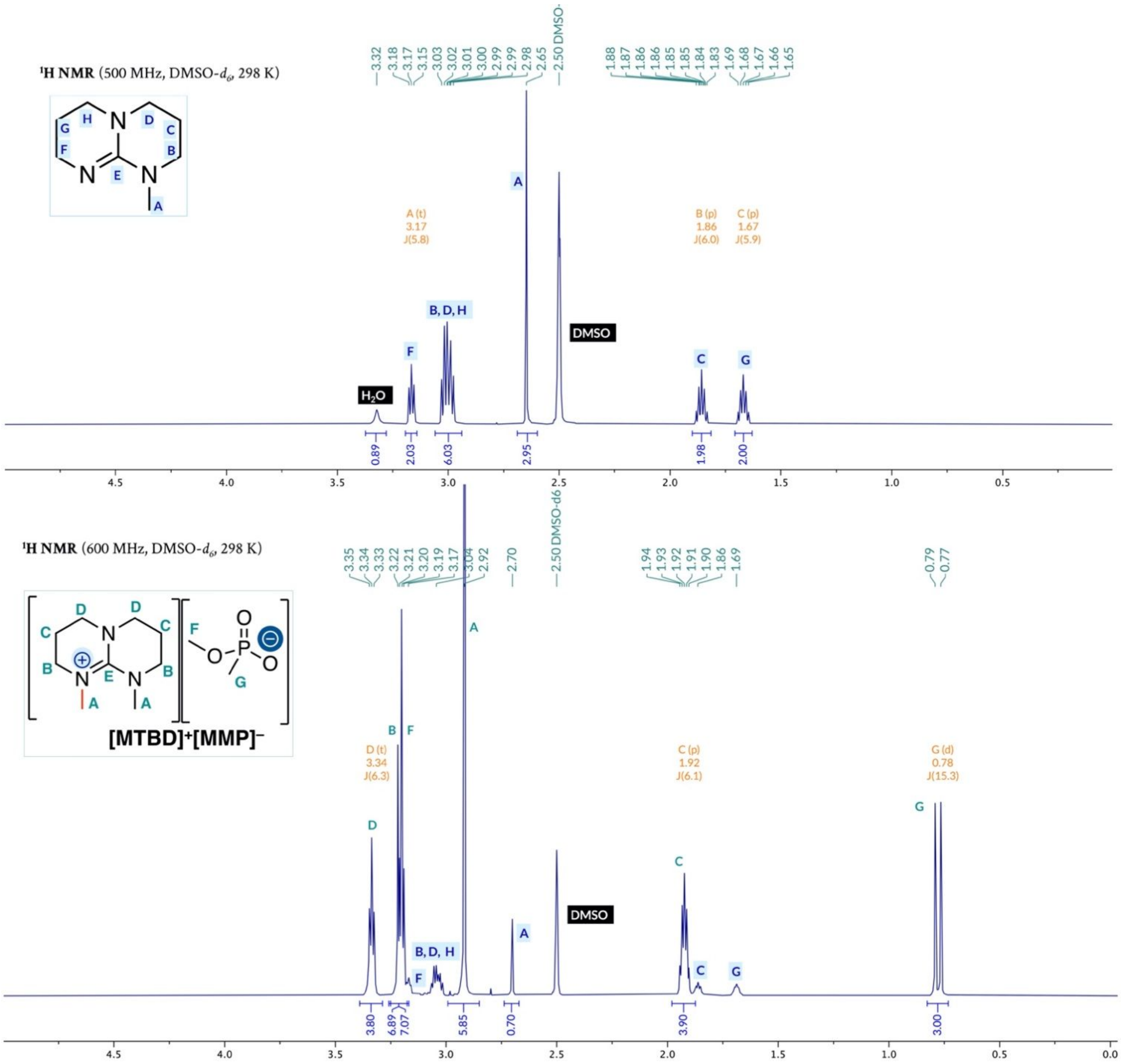

Figure S3. ¹H NMR analysis of the guanidine superbase MTBD and [MTBD]⁺[MMP]⁻

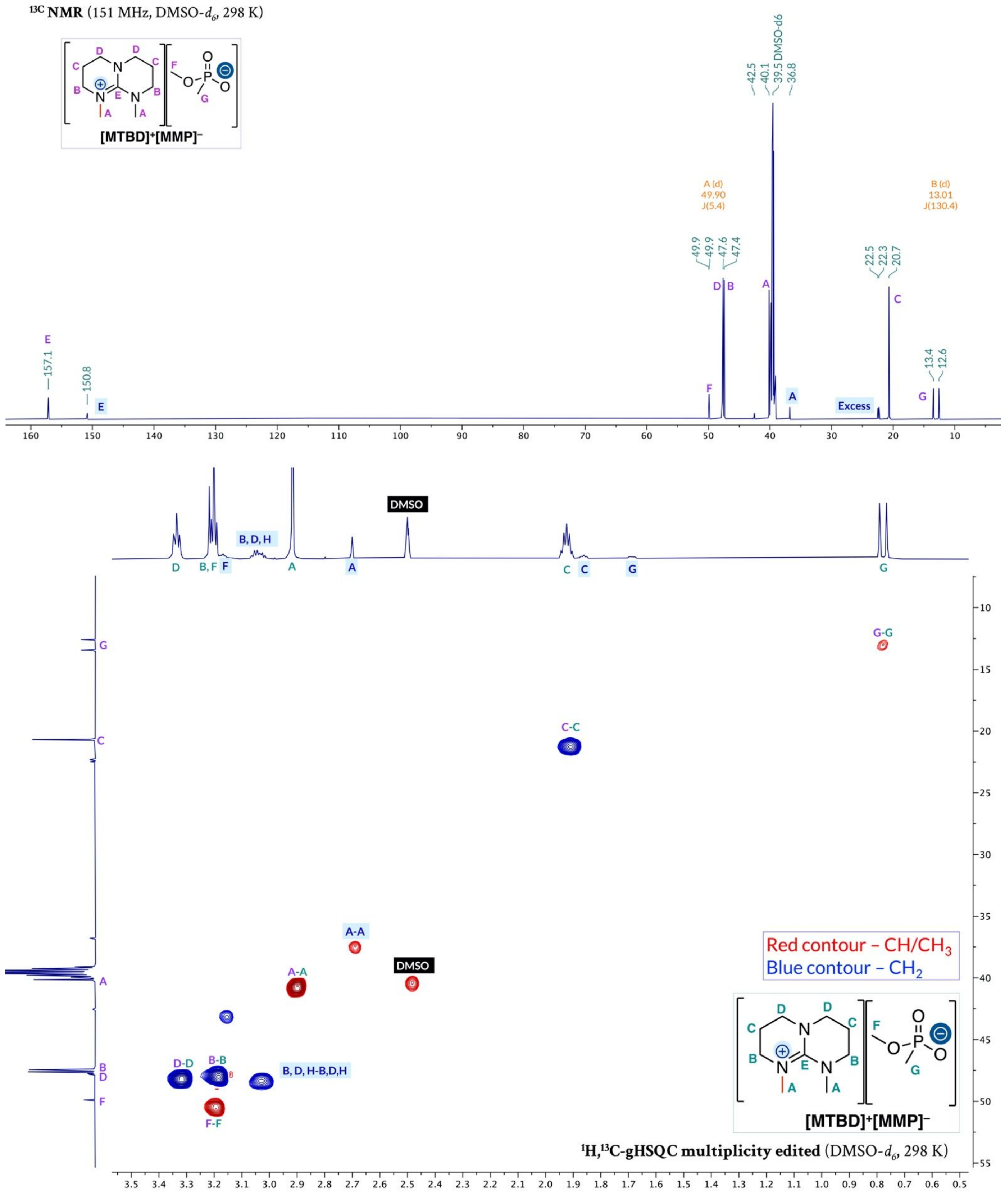


Figure S4. $^{13}C$ NMR analysis of the ionic liquid $[MTBD]^{+}[MMP]^{-}$

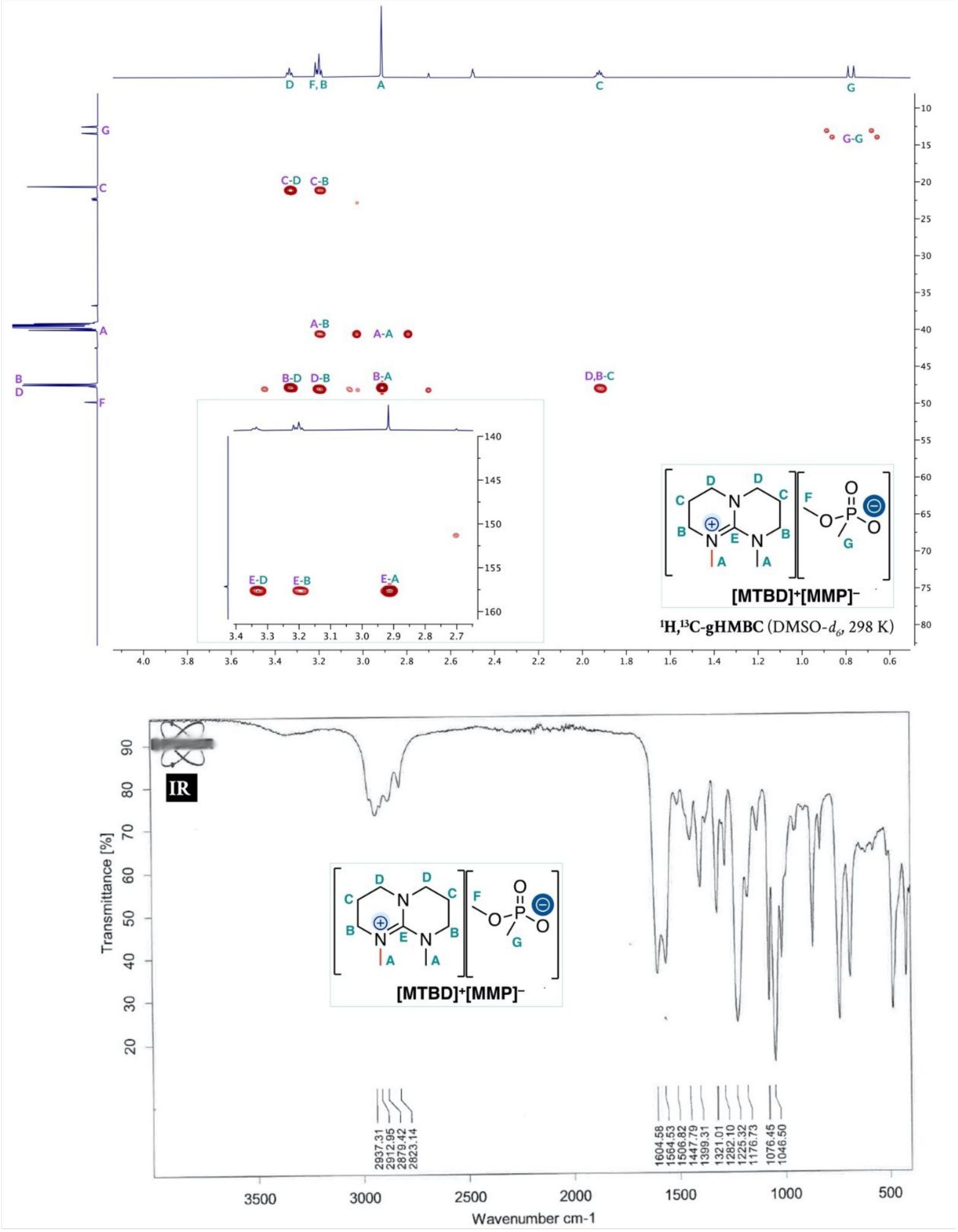


Figure S5. NMR analysis of the ionic liquid [MTBD]+[MMP]− and ATR-IR of [MTBD]+[MMP]−

### 1.2.4. Stability of [MTBD]⁺[MMP]⁻

According to the described procedure, two different batches of the [MTBD]⁺[MMP]⁻ IL were prepared in two different molar ratios: 1) 1.0:1.0 and 2) 1.2:1.0. Both batches were stored and manipulated under Argon to minimize the slow hydrolysis of the guanidine base that occurs upon sustained exposure to moisture. An aliquot of each batch (ca. 20-30 mg.) was taken and dissolved in DMSO-*d6* for $^{1}H$ and $^{31}P$ NMR analysis to evaluate the stability of the IL over time.

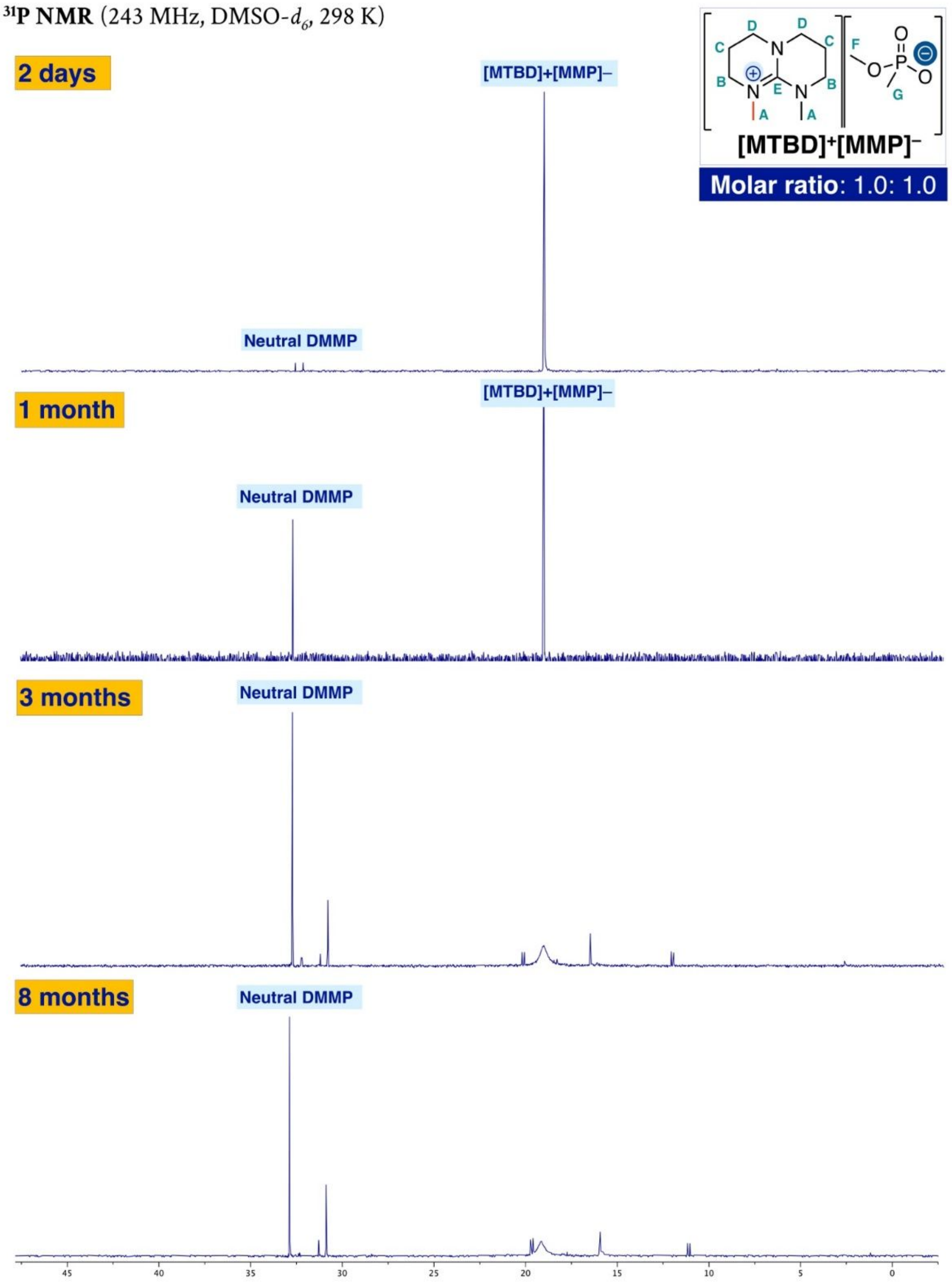


Figure S6. $^{31}P$ NMR, chemical stability analysis of [MTBD]⁺[MMP]⁻ with molar ration 1.0:1.0

$^{31}$**P NMR** (243 MHz, DMSO-$d_6$, 298 K)

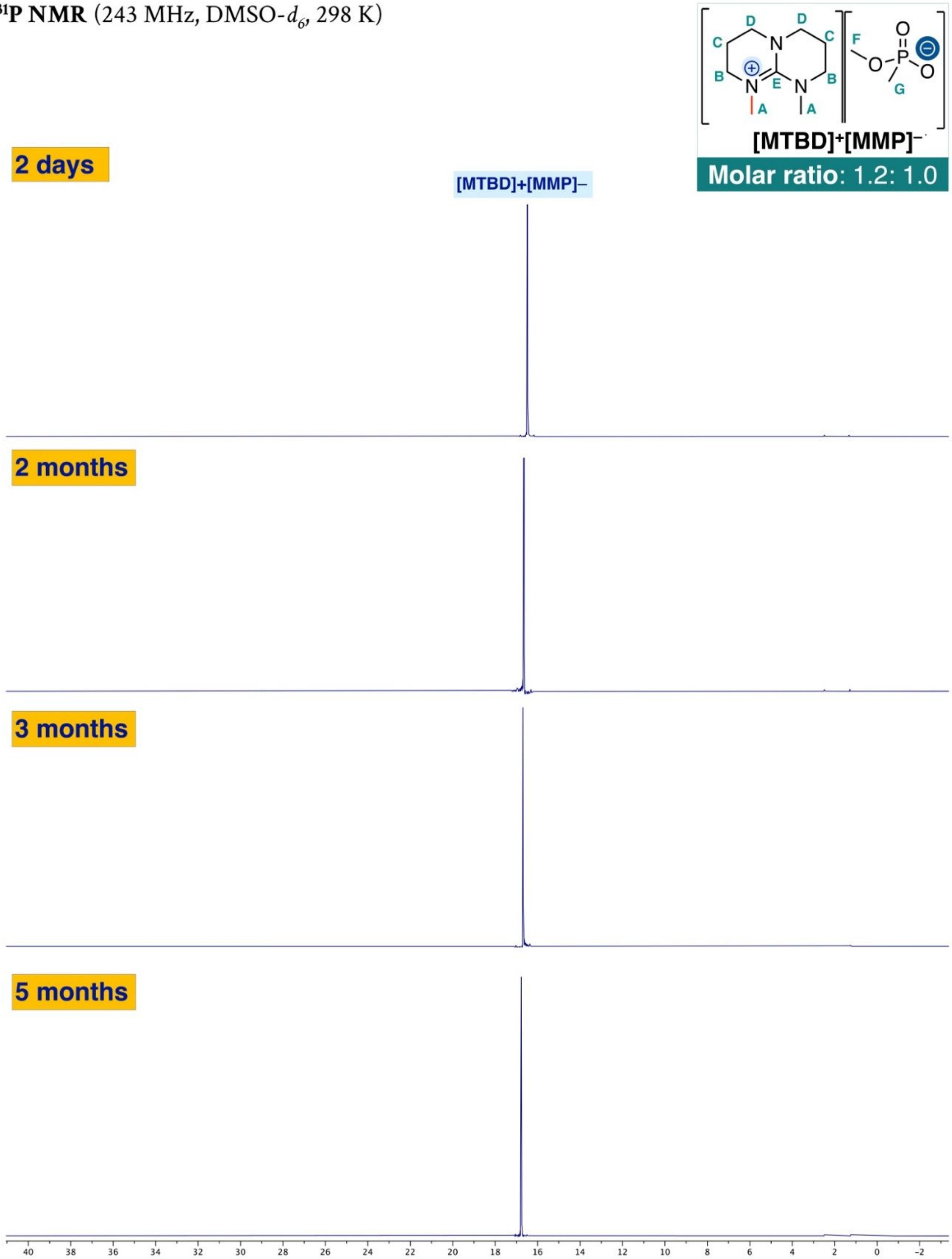


Figure S7. $^{31}$P NMR, chemical stability analysis of $[MTBD]^+[MMP]^-$ with molar ration 1.2:1.0

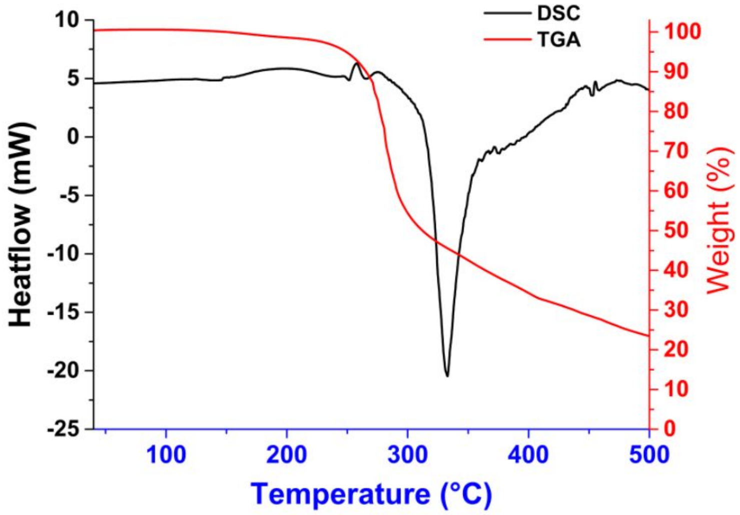


Figure S8. Thermal analysis of the IL [MTBD][MMP]. Black line represents the DSC and red line TGA.

## 2. Aerogel supplementary results

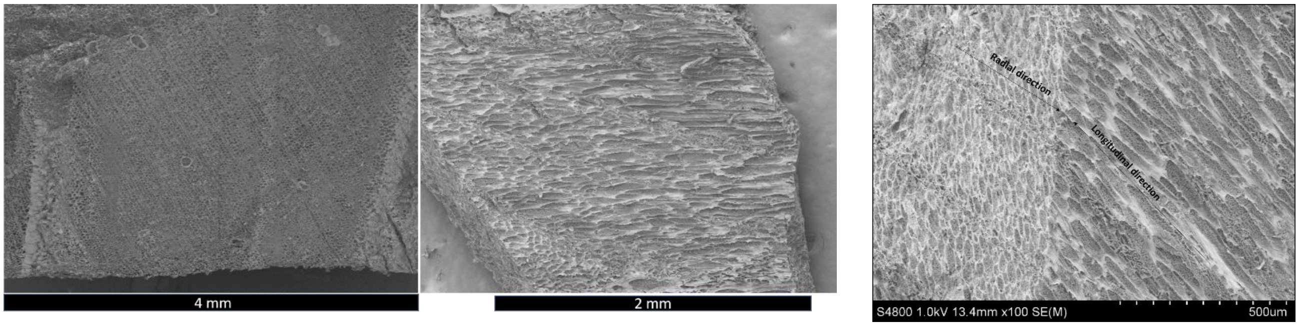


Figure S9. Wood morphology after 24 hours of IL treatment. Here networks are seen to fill every fibre of the wood in radial, transversal and longitudinal direction. Starting from the right hand-side, the remarkable homogeneity is accentuated showing filling of every lumen of the entire wood piece of about 4 mm. The second image portrays the filled fibers from a cut along the fiber direction. The last image shows an intercept of the radial and longitudinal direction.

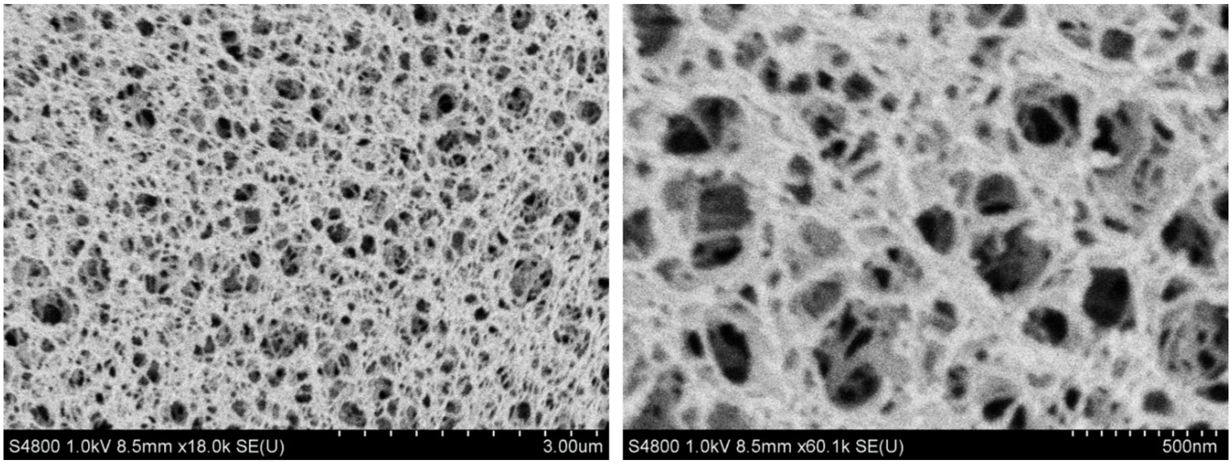


Figure S10. High magnification of lumen fibril network structure from 24h treated aerogels.

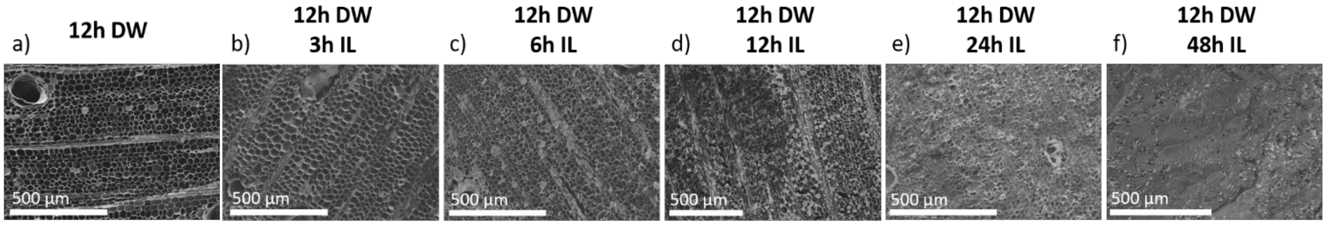


Figure S11. Scanning electron microscope images of cross-section cut a) 12h Delignified wood (DW), and IL treated DW for b) 3h, c) 6h, d) 12h, e) 24h and f) 48h.

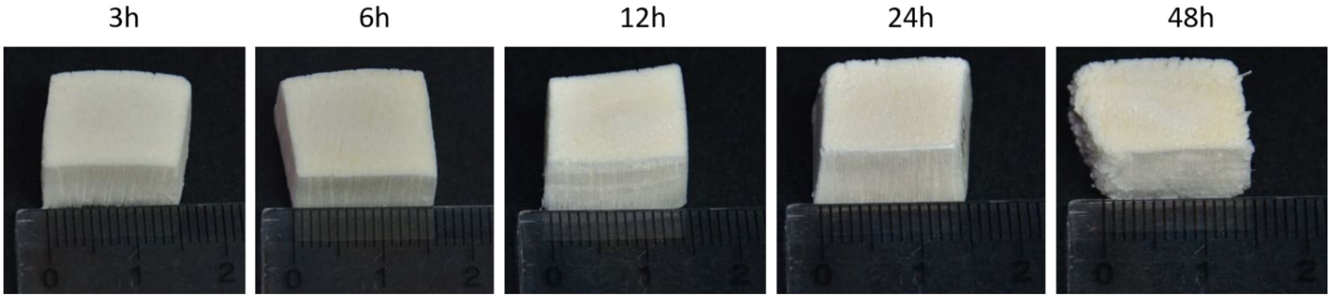


Figure S12. Visual dimensional changes along ionic liquid treatment.

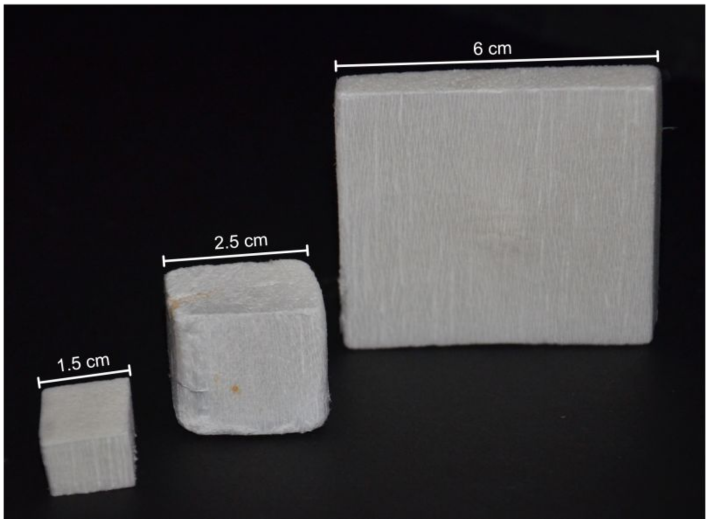


Figure S13. Upscaling of wood aerogels.

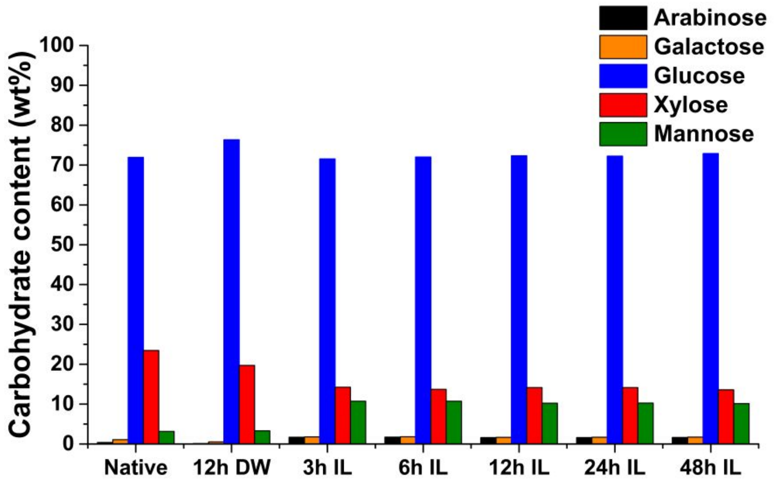


Figure S14. Average carbohydrate content for native wood, delignified wood and all IL treatment times. Glucose monosugars represents cellulose, whereas arabinose, galactose, mannose and xylose are the main constituents in hemicelluloses.

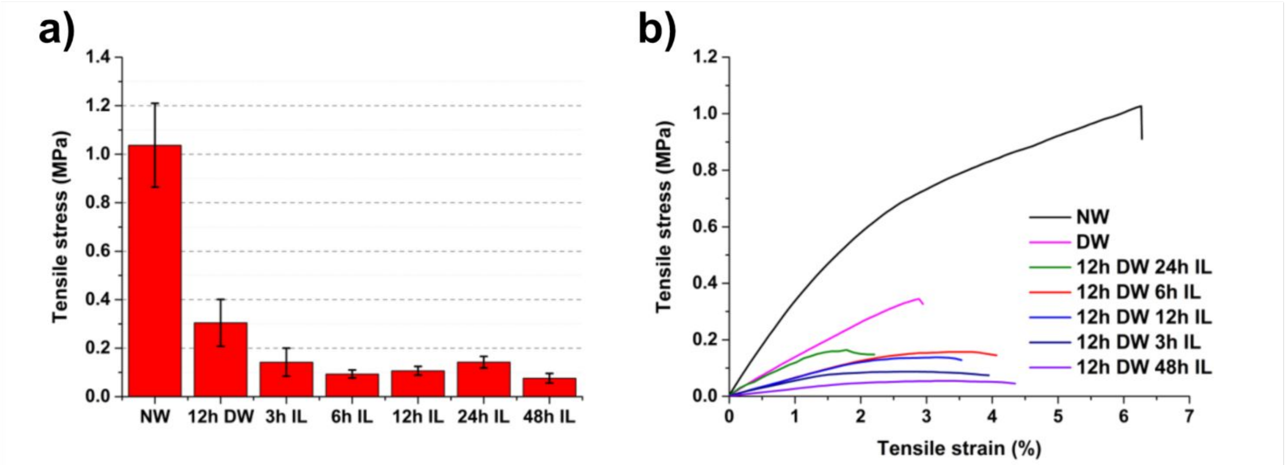


Figure S15. a) Transversal tensile strain of all specimens with standard deviation, b) typical stress-strain curves for each specimen

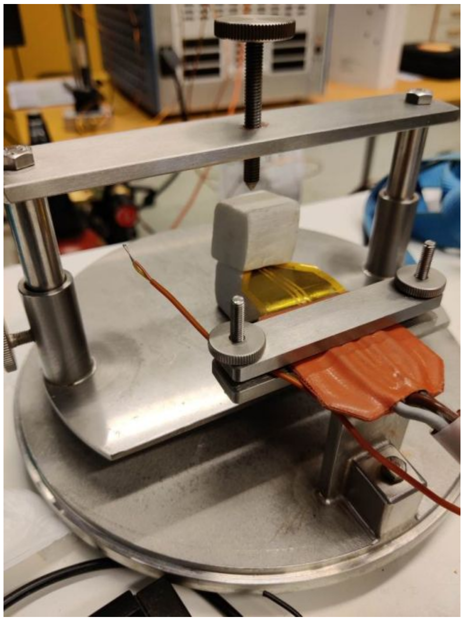

Figure S16. Hot plate thermal conductivity measurements. A hot plate is sandwiched between two aerogels. When in operation, a metal plate is position under the screw to put some load on the samples, to improve the connection with the hot disk. Red cable on the left hand-side is for temperature measurements during the measurements.

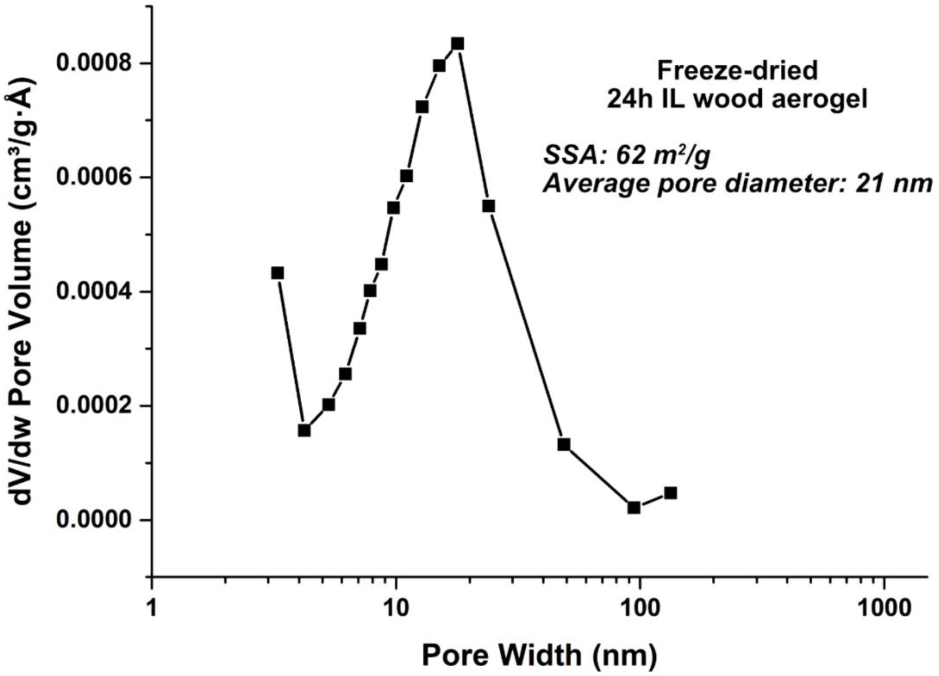


Figure S17. Pore-size distribution of freeze-dried 24h IL treated wood aerogel used for thermal insulation measurements.

## Aerogel - Radial

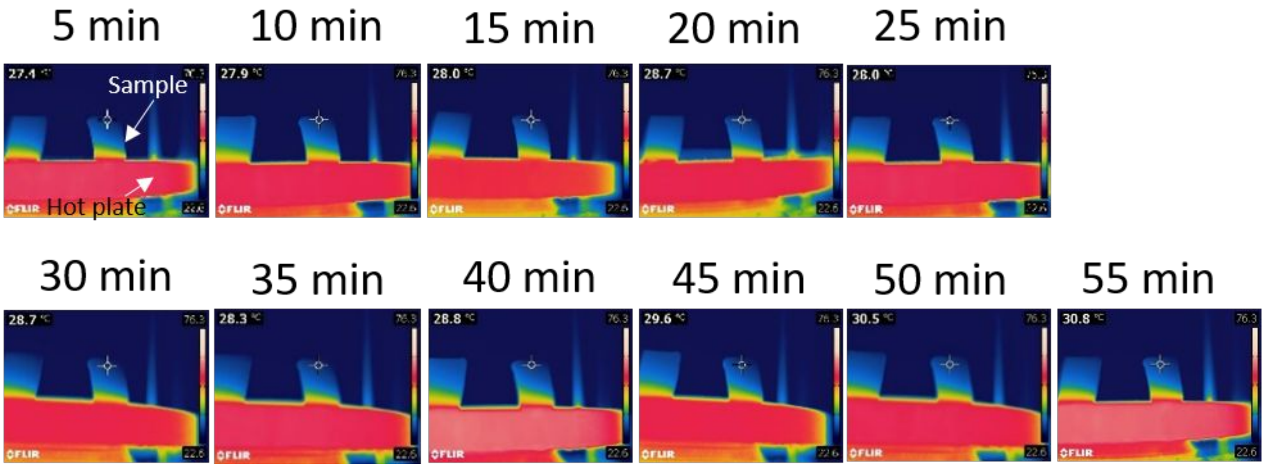


## NW - Radial

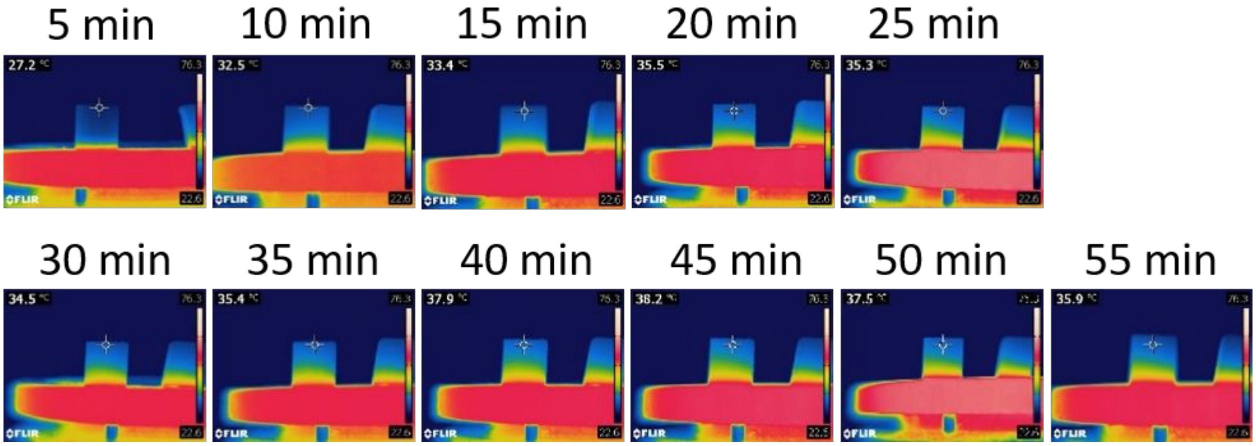

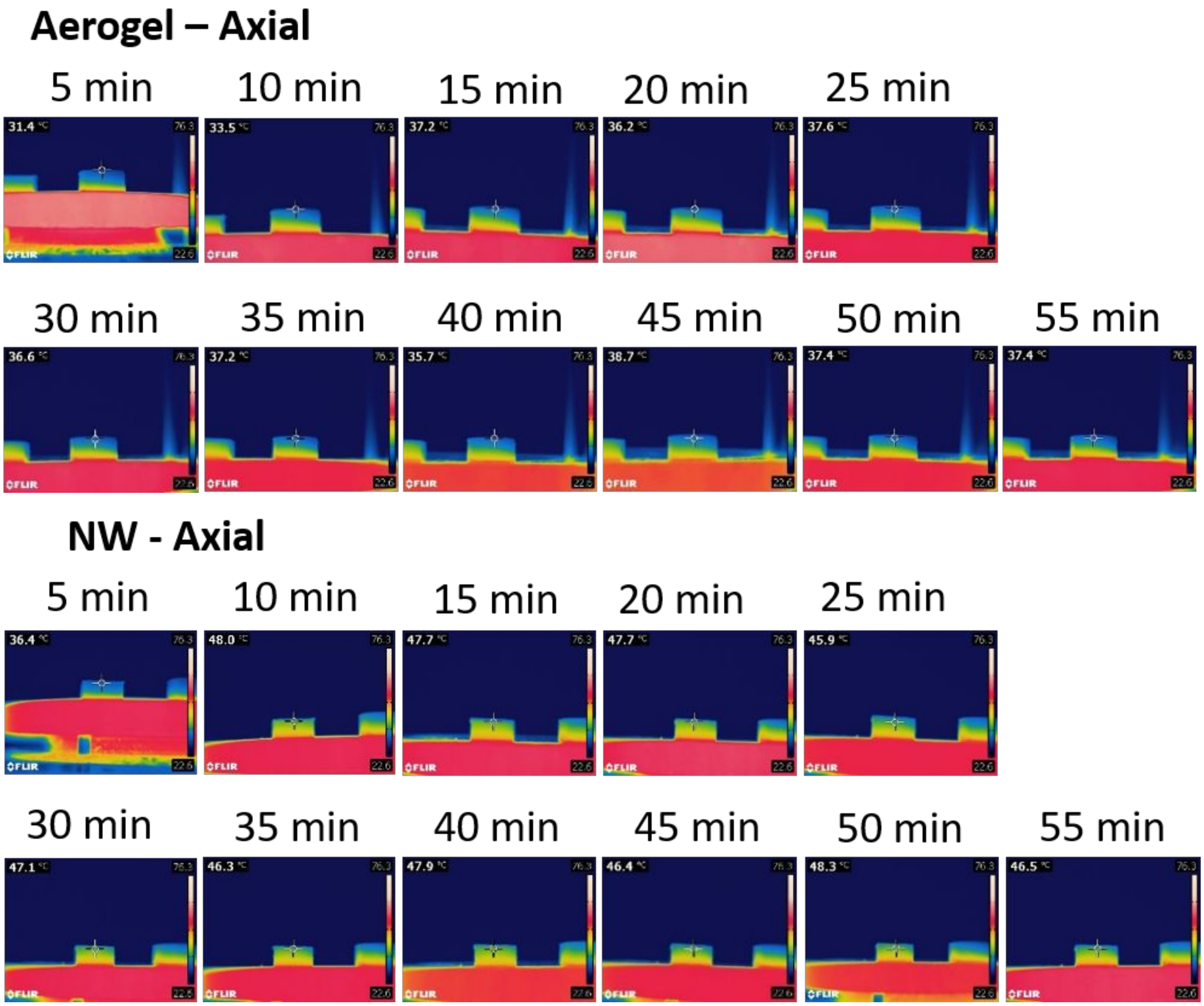


Figure S18. IR-images of each consecutive data point for native wood and wood aerogel in radial and axial direction of the wood. Time and direction are assign to each image in ascending order. The hot plate had a stable temperature of 70 °C.

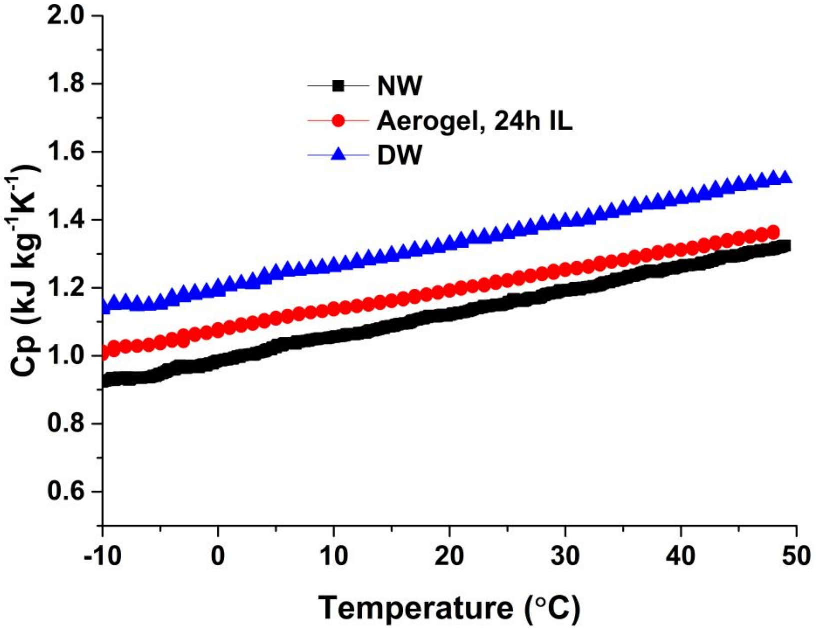


Figure S19. Specific heat capacity of treated samples

Table S1. Density, weight loss and dimensional changes for all treatments of the wood aerogels

| | **Density (kg/m³)** | **Weight loss (%)** | **Width (mm)** | **Length (mm)** | **Thickness (mm)** |
|---|---|---|---|---|---|
| **NW** | 111 ± 10 | 0 | | | |
| **DW** | 77 ± 1 | 18 | 17.6 ± 0.22 | 16.0 ± 0.1 | 10.6 ± 0.1 |
| **3h IL** | 71 ± 6 | 46 | 16.5±0.5 | 13.9 ± 0.2 | 9.2 ± 0.12 |
| **6h IL** | 73 ± 9 | 42 | 17.0 ± 0.4 | 13.6 ± 0.4 | 9.4 ± 0.4 |
| **12h IL** | 74 ± 6 | 47 | 16.2 ± 0.3 | 13.1 ± 0.4 | 9.0 ± 0.2 |
| **24h IL** | 72 ± 6 | 54 | 15.2 ± 0.6 | 13.1 ± 0.7 | 8.8 ±0.1 |
| **48h IL** | 80 ± 3 | 52 | 15.7 ± 0.4 | 11.96 ± 0.2 | 9.0 ± 0.3 |

Table S2. Thermal diffusivity of the tested samples

| | **Thermal diffusivity** | |
|---|---|---|
| | **Axial (mm²/s)** | **Radial (mm²/s)** |
| **NW** | 0.47 ± 0.013 | 0.30 ± 0.003 |
| **DW** | 0.87 ± 0.020 | 0.50 ± 0.051 |
| **Wood aerogel (24h)** | 0.75 ± 0.010 | 0.48 ± 0.021 |

## 3. References


**S1.** Hayes, R.; Warr, G. G.; Atkin, R. Structure and Nanostructure in Ionic Liquids. *Chem. Rev.* **2015**, *115*, 6357–6426.

**S2.** Jiang, H. J.; Atkin, R.; Warr, G. G. Nanostructured Ionic Liquids and Their Solutions: Recent Advances and Emerging Challenges. *Curr. Opin. Green. Sustain. Chem.* **2018**, *12*, 27–32.

**S3**. Brandt, A.; Gräsvik, J.; Hallett, J. P.; Welton, T. Deconstruction of Lignocellulosic Biomass with Ionic Liquids. *Green Chem.* **2012**, *15*, 550–583.

**S4.** Morais, E. S.; Lopes, A. M. da C.; Freire, M. G.; Freire, C. S. R.; Coutinho, J. A. P.; Silvestre, A. J. D. Use of Ionic Liquids and Deep Eutectic Solvents in Polysaccharides Dissolution and Extraction Processes towards Sustainable Biomass Valorization. *Molecules* **2020**, *25*, 3652.

**S5.** Wang, H.; Gurau, G.; Rogers, R. D. Ionic Liquid Processing of Cellulose. *Chem. Soc. Rev.* **2012**, *41*, 1519–1537.

**S6.** Lu, B.; Xu, A.; Wang, J. Cation Does Matter: How Cationic Structure Affects the Dissolution of Cellulose in Ionic Liquids. *Green Chem.* **2013**, *16*, 1326–1335.

**S7.** Fukaya, Y.; Hayashi, K.; Wada, M.; Ohno, H. Cellulose Dissolution with Polar Ionic Liquids under Mild Conditions: Required Factors for Anions. Green Chem. 2008, 10, 44−46.

**S8.** Abe, M.; Fukaya, Y.; Ohno, H. Extraction of Polysaccharides from Bran with Phosphonate or Phosphinate-derived Ionic Liquids under Short Mixing Time and Low Temperature. Green Chem. 2010, 12, 1274−1280.

**S9.** Hirosawa, K.; Fujii, K.; Hashimoto, K.; Shibayama, M. Solvated Structure of Cellulose in a Phosphonate-Based Ionic Liquid. *Macromolecules* **2017**, *50*, 6509–6517.

**S10.** Hokayem, K. A.; Hage, R. E.; Svecova, L.; Otazaghine, B.; Moigne, N. L.; Sonnier, R. Flame Retardant-Functionalized Cotton Cellulose Using Phosphonate-Based Ionic Liquids. *Molecules* 2020, *25*, 1629.

**S11.** Egorova, K. S.; Gordeev, E. G.; Ananikov, V. P. Biological Activity of Ionic Liquids and Their Application in Pharmaceutics and Medicine. *Chem. Rev.* **2017**, *117*, 7132–7189.

**S12.** Flieger, J.; Flieger, M. Ionic Liquids Toxicity-Benefits and Threats. *Int. J. Mol. Sci.* **2020**, *21*, 6267.

**S13.** Ebner, G.; Schiehser, S.; Potthast, A.; Rosenau, T. Side Reaction of Cellulose with Common 1-Alkyl-3-Methylimidazolium-Based Ionic Liquids. *Tetrahedron Lett.* **2008**, *49*, 7322–7324.

**S14.** Clough, M. T.; Geyer, K.; Hunt, P. A.; Son, S.; Vagt, U.; Welton, T. Ionic Liquids: Not Always Innocent Solvents for Cellulose. *Green Chem.* **2014**, *17*, 231–243.

**S15**. Cerro, D. R. del; Mera-Adasme, R.; King, A. W. T.; Perea-Buceta, J. E.; Heikkinen, S.; Hase, T.; Sundholm, D.; Wähälä, K. On the Mechanism of the Reactivity of 1,3-Dialkylimidazolium Salts under Basic to Acidic Conditions: A Combined Kinetic and Computational Study. *Angew. Chem. Int. Ed.* **2018**, *57*, 11613–11617.

**S16**. Gao, Y.; Arritt, S. W.; Twamley, B.; Shreeve, J. M. Guanidinium-Based Ionic Liquids. *Inorg. Chem.* **2005**, *44*, 1704–1712.

**S17**. King, A. W. T.; Asikkala, J.; Mutikainen, I.; Järvi, P.; Kilpeläinen, I. Distillable Acid–Base Conjugate Ionic Liquids for Cellulose Dissolution and Processing. *Angew. Chem. Int. Ed.* **2011**, *50*, 6301–6305.

**S18.** Asaadi, S.; Kakko, T.; King, A. W. T.; Kilpeläinen, I.; Hummel, M.; Sixta, H. High-Performance Acetylated Ioncell-F Fibers with Low Degree of Substitution. *ACS Sustain. Chem. Eng.* **2018**, *6*, 9418–9426.

**S19.** Parviainen, A.; Wahlström, R.; Liimatainen, U.; Liitiä, T.; Rovio, S.; Helminen, J. K. J.; Hyväkkö, U.; King, A. W. T.; Suurnäkki, A.; Kilpeläinen, I. Sustainability of Cellulose Dissolution and Regeneration in 1,5-Diazabicyclo[4.3.0]Non-5-Enium Acetate: A Batch Simulation of the IONCELL-F Process. *RSC Adv.* **2015**, *5*, 69728–69737.

**S20.** Holding, A. J.; Heikkilä, M.; Kilpeläinen, I.; King, A. W. T. Amphiphilic and Phase-Separable Ionic Liquids for Biomass Processing. *ChemSusChem* **2014**, *7*, 1422–1434.

**S21.** Efficiency of Hydrophobic Phosphonium Ionic Liquids and DMSO as Recyclable Cellulose Dissolution and Regeneration Media. *RSC Adv.* **2017**, *7*, 17451–17461.

**S22.** Cerro, D. R. del; Koso, T. V.; Kakko, T.; King, A. W. T.; Kilpeläinen, I. Crystallinity Reduction and Enhancement in the Chemical Reactivity of Cellulose by Non-Dissolving Pre-Treatment with Tetrabutylphosphonium Acetate. *Cellulose* **2020**, *27*, 5545–5562.
